# High Risks of Losing Genetic Diversity in an Endemic Mauritian Gecko: Implications for Conservation

**DOI:** 10.1371/journal.pone.0093387

**Published:** 2014-06-25

**Authors:** Steeves Buckland, Nik C. Cole, Jim J. Groombridge, Clemens Küpper, Terry Burke, Deborah A. Dawson, Laura E. Gallagher, Stephen Harris

**Affiliations:** 1 School of Biological Sciences, University of Bristol, Bristol, United Kingdom; 2 NERC Biomolecular Analysis Facility, Department of Animal and Plant Sciences, University of Sheffield, Sheffield, United Kingdom; 3 National Parks & Conservation Service, Reduit, Mauritius; 4 Durrell Wildlife Conservation Trust, Jersey, Channel Islands; 5 Mauritian Wildlife Foundation, Vacoas, Mauritius; 6 Durrell Institute of Conservation and Ecology, School of Anthropology and Conservation, University of Kent, Kent, United Kingdom; Instituto de Higiene e Medicina Tropical, Portugal

## Abstract

Genetic structure can be a consequence of recent population fragmentation and isolation, or a remnant of historical localised adaptation. This poses a challenge for conservationists since misinterpreting patterns of genetic structure may lead to inappropriate management. Of 17 species of reptile originally found in Mauritius, only five survive on the main island. One of these, *Phelsuma guimbeaui* (lowland forest day gecko), is now restricted to 30 small isolated subpopulations following severe forest fragmentation and isolation due to human colonisation. We used 20 microsatellites in ten subpopulations and two mitochondrial DNA (mtDNA) markers in 13 subpopulations to: (i) assess genetic diversity, population structure and genetic differentiation of subpopulations; (ii) estimate effective population sizes and migration rates of subpopulations; and (iii) examine the phylogenetic relationships of haplotypes found in different subpopulations. Microsatellite data revealed significant population structure with high levels of genetic diversity and isolation by distance, substantial genetic differentiation and no migration between most subpopulations. MtDNA, however, showed no evidence of population structure, indicating that there was once a genetically panmictic population. Effective population sizes of ten subpopulations, based on microsatellite markers, were small, ranging from 44 to 167. Simulations suggested that the chance of survival and allelic diversity of some subpopulations will decrease dramatically over the next 50 years if no migration occurs. Our DNA-based evidence reveals an urgent need for a management plan for the conservation of *P. guimbeaui*. We identified 18 threatened and 12 viable subpopulations and discuss a range of management options that include translocation of threatened subpopulations to retain maximum allelic diversity, and habitat restoration and assisted migration to decrease genetic erosion and inbreeding for the viable subpopulations.

## Introduction

The effects of habitat fragmentation on genetic structure are well documented [1]. Disconnected habitat fragments harbour small, isolated populations [2], which can lead to loss of genetic diversity, inbreeding depression and reduced levels of population-wide fitness [3]–[5], all factors that can increase the risk of extinction [6]. Reduced genetic diversity following habitat fragmentation [7], combined with low dispersal [8], can also limit the ability of populations to adapt to environmental change [6]. Therefore, a detailed knowledge of how population genetic diversity is structured across fragmented landscapes, and the extent of genetic differentiation, connectivity and effective population sizes (N_e_), are key to formulating a conservation strategy that maintains genetic variability and promotes the evolutionary potential of threatened species [9], [10].

Understanding patterns of population genetic structure frequently poses a challenge to conservation managers. It is important to determine whether the observed genetic structure is a consequence of recent population fragmentation and isolation, or a remnant of historical localised adaptation. Misdiagnosing the former when the latter is true risks disrupting patterns of local adaptation and outbreeding depression if incompatible populations are mixed. Conversely, interpreting structured patterns to be signatures of local adaptation when they are a consequence of isolation and drift risks inappropriate management to maintain existing genetic patterns, when maximising gene flow between populations might reduce genetic loss and the risk of extinction. Identifying the origins of genetic structure is particularly important when deciding whether to use translocation to reinforce existing populations and/or establish new populations, and to determine how many founding individuals are required to retain the existing genetic diversity. It is also important to interpret genetic patterns alongside ecological factors such as habitat loss when deciding on the most appropriate management option.

In Mauritius, successive occupations by the Dutch (1638–1710), French (1721–1810) and British (1810–1968) have destroyed a large part of the ecosystem through habitat destruction and the introduction of invasive alien species [11]; over this period, Mauritius has experienced one of the highest known rates of extinction in the world [11]. Of the 17 described species that once formed a rich endemic terrestrial reptile community [12], only five remain on mainland Mauritius (five are extinct, the other seven only survive on small offshore islands). Four of the species still found on the mainland are day geckos (*Phelsuma*), and these support a wide range of ecological functions such as pollination, predator-prey dynamics and seed dispersal [11]. *Phelsuma guimbeaui* (lowland forest day gecko) is the species most vulnerable to extinction. It persists in only 30 small and isolated subpopulations, many of which may be lost within the next decade due to increasing urbanisation, habitat loss, and the impact of invasive species such as *Phelsuma grandis* (giant Madagascar day gecko), introduced to Mauritius in the 1990s (Buckland et al., submitted).

Here, we characterise genetic diversity and structure for *P. guimbeaui* and consider the most appropriate strategy for its long-term conservation. We used a suite of microsatellite markers for *P. guimbeaui* [13] and two mtDNA markers to: (i) quantify levels of genetic diversity, the extent of population structure and genetic differentiation within and between subpopulations; (ii) estimate effective population size for each subpopulation and the degree of gene flow (migration); and (iii) examine the phylogenetic relationships of haplotypes among the different subpopulations. We evaluated whether the levels of genetic structure and diversity reflected recent or ancestral patterns, and interpret our findings in the light of the well-documented chronology of habitat loss recorded in Mauritius by the early European colonists. We then used simulations to: (iv) estimate the probability of survival and retaining rare alleles in a subpopulation; and (v) estimate the number of individuals that should be translocated initially from a subpopulation, and the number of geckos that need to be translocated at timed intervals thereafter, to form a new population without loss of genetic variation. Finally, we make recommendations for the short- and long-term genetic management of *P. guimbeaui*.

## Methods

### Ethical statement

The capture and tissue sampling were approved by the University of Bristol's Ethical Review Committee (University Investigation Number UB/11/031) and the National Parks and Conservation Service, Ministry of the Agro-Industry, Mauritius.

### Study sites and field methods

**Figure 1:**
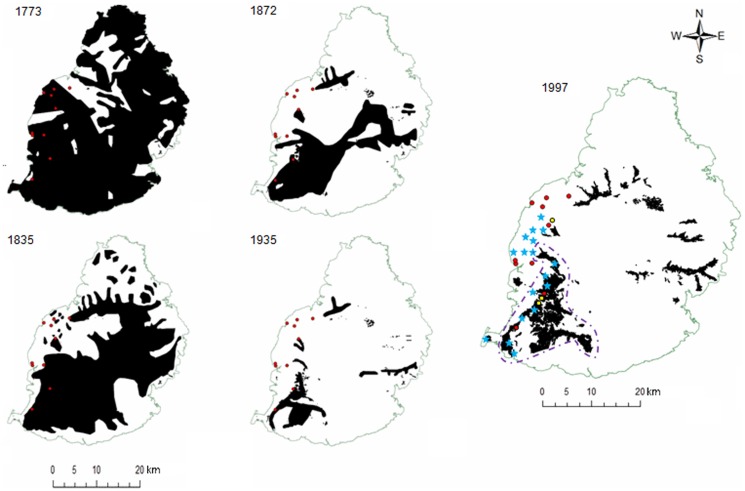
Pattern of deforestation in Mauritius from 1773 to 1997. The red dots indicate the 10 subpopulations for which both microsatellite and mtDNA analyses were conducted, and the yellow dots the three subpopulations for which only mtDNA analyses were carried out. The blue stars mark subpopulations not sampled and the black region within the purple dotted line on the 1997 map shows the Black River mountains. All subpopulation locations were transposed by 1

For the purpose of this study, we defined a subpopulation as inhabiting a piece of wooded habitat separated by barriers such as a major road, large area of agriculture, non-habitable planted/non-planted exotic trees such as *Pinus taeda* (loblolly pine) and *Psidium cattleianum* (strawberry guava), or human habitation. Between 2007 and 2009, we searched the western part of Mauritius and found a total of 30 subpopulations (Figure 1) covering areas ranging in size from 0.006 to 1 km^2^. Twelve of the 30 subpopulations were in the Black River mountains in high-quality native forest (>90% native cover).

**Figure 2:**
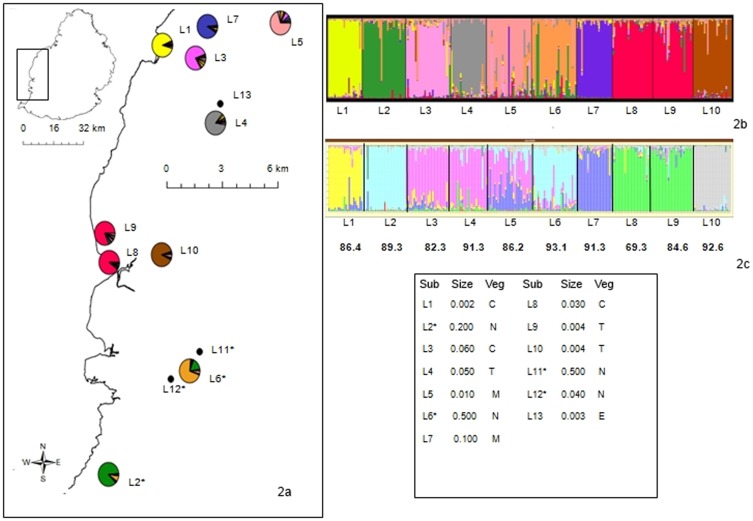
Location and assignment to genetic clusters of ten subpopulations of *Phelsuma guimbeaui*. **a** Subpopulations used for the microsatellite analyses. Colours in the pie charts indicate the proportion of genetic clusters identified using STRUCTURE 2.3.2. The three subpopulations only used for mtDNA analyses (L11, L12 and L13) are also shown in Figure 2a; subpopulations marked with an asterisk are in the Black River mountains. **b** Bar plots showing the genetic identity of individual samples generated using STRUCTURE 2.3.2. **c** Bar plot output from TESS with each subpopulation's labelling assignment (%) from GENECLASS2 shown below. The box gives details of each subpopulation (Subpop): vegetation (Veg) was classified as exotic campeche forest (C), exotic eucalyptus forest (E), exotic mango orchard (M), native forest (N) and exotic terminalia forest (T); size is the area (km^2^) occupied by each subpopulation.

From 17 January to 2 September 2011, samples were collected from 13 accessible subpopulations for which we could obtain permission. Geckos were captured by hand or with a nylon noose on the end of a telescopic pole. The tail tip (∼5 mm) was removed with a sterile scalpel blade, placed in a labelled 1.5 ml microfuge tube containing 94% alcohol and stored at −20°C. We only caught one gecko in subpopulation L11 and three each in L12 and L13 (Figure 2); these were not included in the microsatellite analyses. Sample sizes in the other ten subpopulations ranged from 29 to 37. The nearest-neighbour distances among these ten subpopulations ranged from 0.6 to 27.3 km, and the area of each subpopulation ranged from 0.002 to 0.5 km^2^ (Figure 2).

### DNA extraction and microsatellite amplification

DNA was extracted using an ammonium acetate salt precipitation protocol [14]. The quality and integrity of the DNA was evaluated by gel electrophoresis. DNA concentration was quantified using a Fluostar OPTIMA (Bmg Labtech Ltd, Aylesbury, UK).

We selected 41 polymorphic microsatellite loci with high amplification success, developed specifically for *P. guimbeaui* [13]. We amplified 312 samples in 2 µl multiplex polymerase chain reactions (PCRs) comprising *ca* 10 ng DNA, 1 µl Qiagen Master Mix (Qiagen Ltd, Manchester, UK) [15], 0.2 µM reverse primer, 0.2 µM forward fluorescent primer, and covered by mineral oil. PCRs were conducted with touchdown cycling conditions detailed in [13]. The resulting fragments were sized using an ABI 3730 48-well capillary DNA Analyser (Applied Biosystems, Foster City, CA, USA) and genotypes scored in GENEMAPPER v3.7 (Applied Biosystems, Foster City, CA, USA). The 41 loci were subsequently subjected to an elimination process whereby unsuitable loci were dropped from further analyses (see Results).

### Microsatellite analyses

Forty samples were randomly selected, re-PCRed and genotyping error rates per allele [16] estimated using MICROSATELLITE TOOLKIT [17]. Large allelic dropout and stutter-band scoring errors were investigated in MICRO-CHECKER v2.2.3 [18]. We used ML-RELATE [19] to identify 55 full- and half-sib relationships, which were excluded since their inclusion could bias the population structure results [20]. We tested whether microsatellite loci were under selection using LOSITAN [21] and subsequently removed any markers so identified. All loci were tested for deviation from Hardy-Weinberg (HW) equilibrium and all pairs of loci tested for linkage disequilibrium (LD) in each subpopulation using GENEPOP v4.0.10 [22]. False discovery rate [23] was used to correct P values in multiple tests. The null allele frequency per locus was estimated using CERVUS v3.0 [24].

To detect genetic diversity within each subpopulation, the number of different alleles, mean number of different alleles (N_A_), allelic richness (A_R_) and private allelic richness (P_A_) were calculated using a rarefaction approach in the software HP-RARE [25]. The mean observed (H_O_) and expected heterozygosities (H_E_) for each subpopulation were estimated in CERVUS v3.0 and the mean inbreeding coefficient (F_IS_) was calculated using GENETIX v4.05 (http://kimura.univ-montp2.fr/genetix/), based on 10,000 permutations. Differences in A_R_ and H_E_ were examined using a parametric ANOVA conducted in R 2.15.3 (R Development Core Team 2013).

Three different analyses were used to assess population structure. First, the Bayesian clustering software STRUCTURE v2.3.2 [26] was used. This uses a Markov Chain Monte Carlo (MCMC) approach to assign individuals to their most likely genetic cluster (K) and we used the admixture model with correlated allele frequencies [27]. Ten independent runs of 1,000,000 MCMC iterations, each with a burn-in of 500,000 generations, were explored. No prior information was provided on the geographical locations of samples [26]. The model with the highest log posterior probability Pr (X|K) [26] and highest delta K [28] was examined to identify the most likely value of K. These two parameters were computed using the online version of STRUCTURE HARVESTER [29]. CLUMMP v1.1.2 [30] was then used to infer the global cluster membership (Q) by averaging the results from the independent runs. Clusters were visualised as bar plot charts in DISTRUCT v1.1 [31]. Second, we used TESS [32] to infer the degree of population structure. TESS uses spatial information regarding the location of each individual together with its genotype. For each K value (2–10), an admixture model with ten independent runs of 100,000 sweeps and a burn-in of 20,000 was performed. The lowest deviance information criterion (DIC) was used to choose the best-fitting model. Third, we used the Bayesian assignment method in GENECLASS2 [33] and population assignment was conducted as in [34] with type 1 error set to 0.05, using 1,000 iterations and simulation computation [35].

Weir and Cockerham's F_st_ [36] values were used to assess population genetic differentiation: a pairwise F_st_ matrix was computed and statistical significance estimated with a permutation test of 9,999 replicates in GenAlEx 6.5 [37]. A hierarchical Analysis of Molecular Variance (AMOVA) [38] was used to establish the major sources of genetic variation. The statistical significance of the AMOVA was assessed with 9,999 permutations in GenAlEx 6.5. The software SPAGeDi v1.3 [39] was used to investigate patterns of isolation by distance (IBD) [40]: this was visualised by plotting pairwise genetic distances (F_st_/(1-F_st_) against the log-transformed geographical distances among the ten subpopulations.

To determine whether levels of genetic diversity (i.e. A_R_ and H_E_) were related to patterns of habitat loss and isolation, we compiled data on forest cover between 1773 and 1935 from [41] and in 1997 from [42]. The habitat maps were then digitised in ARC GIS 10.1 (ESRI, Redlands, CA, USA). Only the 1997 map was accurately georeferenced as the others were originally hand drawn in the 1940s from historical records of logging and cane production (Figure 1). We then tested whether genetic diversity was related to the time for which subpopulations had been isolated using Pearson's coefficient of correlation in R 2.15.3. We assumed that the loss of native habitats prevented natural migration and eventually reduced genetic diversity. *P. guimbeaui* is a habitat specialist and is mostly reliant on native habitats that have high tree diversity and tall, large trees with cavities (Buckland et al., submitted). The maximum dispersal distance of 28 adult *P. guimbeaui* monitored for a year was <15 m (S. Buckland, unpublished data). There are no data on dispersal in sub-adult and juvenile *P. guimbeaui*, but the maximum recorded dispersal distance for *P. ornata* (ornate day gecko), a similar-sized Mauritian species, was 87 m [43]. While *P. guimbeaui* also persists in the presence of four exotic tree species, i.e. *Eucalyptus tereticornis* (eucalyptus), *Haematoxylum campechianum* (campeche), *Mangifera indica* (mango) and *Terminalia arjuna* (terminalia), these trees are usually scattered and probably act as ecological traps; there are no data on the density of *P. guimbeaui* in these exotic plantations, and it has never been recorded on buildings or in agricultural land.

### Effective population size and contemporary migration

N_e_ was estimated using two different techniques. First, we used a point estimate approach using LD in N_e_ ESTIMATOR [44]. Second, a coalescent computation as implemented in MIGRATE [45] was used, in which the following settings were applied: slice sampling with uniform theta prior boundaries of 0 and 100; a Brownian microsatellite model and constant mutation rate; four static chains (default temperatures); and Watterson estimator theta initially estimated from F_st_. We carried out four independent runs with 5,000,000 iterations after an initial burn-in of 1,000,000 iterations. The software BOTTLENECK v1.2.02 [46] was also used to detect any recent decline in N_e_. We used a general vertebrate multiple step mutation rate (p_g_ = 0.22) [10] and a reptile multiple step mutation rate (p_g_ = 0.46) [47].

Three different techniques were used to estimate contemporary rates of migration between the subpopulations. First, we used a Bayesian approach implemented in BAYESASS v1.3 [48]. The number of iterations was set at 20,000,000, with an initial burn-in of 5,000,000 and a thinning of 2,000 chains. The delta value for allele frequency and inbreeding was kept at the default value of 0.15 and migration was changed to 0.1. Five independent runs were conducted to test for convergence and consistent results. Second, we used GENECLASS2 to detect first-generation migrants. We used the simulation algorithm in [35] and specified L_home as the likelihood criterion, where the number of simulated individuals and type 1 error were set at 10,000 and 0.05, respectively. Third, prior information about the locations of individuals was supplemented using the USEPOPINFO model and MIGRPRIOR was set at 0.001 to output estimates of migration between subpopulations in STRUCTURE v2.3.2 [49].

### Estimating risks of extinction and retention of rare alleles

We first used simulations in the R package AlleleRetain [50] to estimate the probability of survival and retaining rare alleles in each subpopulation. We also estimated the number of individuals that would need to be translocated per subpopulation to retain the maximum number of rare alleles. We used the estimated size of each subpopulation, obtained by multiplying N_e_ by ten [51], and life-history information such as age of maturity and mating system from field and captive data (N. Cole, R. Budzinski and S. Budzinski, unpublished data). The R codes with history information for the simulations are given in Table S1. To evaluate different possible interventions to minimise the risks of extinction and capturing the maximum number of rare alleles in a translocated population, we investigated the following scenarios: (i) the initial number of translocated individuals; (ii) the number of assisted migrants after translocation; and (iii) the frequency at which assisted migrants were translocated, where assisted migrants were geckos translocated in subsequent years after the initial translocation, and the frequency at which assisted migrants were translocated is the number of years after the initial translocation when assisted migrants were translocated. All simulations were conducted across 1,000 replications over a 50-year period.

### Mitochondrial sequencing

Partial regions of *cyt b* and *16S rRNA*, two mitochondrial genes frequently used in phylogenetic research [52], were amplified in a 10 µl reaction mixture containing 10 ng of DNA, 5 µl of Mytaq HS DNA Polymerase Mix (Bioline Reagents Ltd., London, UK) and 4 µM of each primer. Primers were designed for mtDNA loci in Primer3 [53] (Table S2). Cycling parameters consisted of an initial denaturation at 95°C for 60 s and 34 cycles starting with denaturation at 95°C for 15 s, annealing at 59°C for 15 s and final extension at 72°C for 10 s. The presence of amplified product was confirmed by visualising a fraction of the product on an agarose gel. PCR products were cleaned up with Exo-SAP-IT (Amersham Biosciences, Piscataway, NJ, USA), precipitated with ethanol and sequenced using the Big Dye Terminator v3.1 Cycle Sequencing Kit on an ABI 3730 DNA Analyser (Applied Biosystems, Foster City, CA, USA).

### Mitochondrial DNA analyses

The start and end of sequences were trimmed. A consensus sequence was obtained by aligning the forward and reverse sequence for each individual in CodonCode Aligner (CodonCode Corporation, Dedham, MA, USA) and complementary alignment was conducted in MEGA v5.05 [54] using the ClustalW algorithm. Mitochondrial DNA sequences were concatenated in Geneious (Biomatters Ltd, Auckland, New Zealand). To quantify the genetic variation in the concatenated sequences, haplotype diversity (H) and nucleotide diversity (π) [55] were calculated using DnaSP v5.10.01 [56]. AMOVA and F_st_ were calculated in GenAlEx 6.5; analyses were performed separately per subpopulation (one to eight individuals) and for the entire population.

Phylogenetic trees of different haplotypes were inferred by applying the maximum-likelihood method in MEGA 5.05 [54] and a Bayesian approach in MRBAYES [57]. MODELTEST v3.7 [58] was used to find the best-fit model of evolution according to the corrected Akaike information criterion (AICc): models were GTR + G and HKY + G for *cyt b* and *16S rRNA*, respectively. For maximum likelihood, a phylogenetic tree with 1,000 bootstraps was used to explore the robustness of tree topology. Bayesian analysis was conducted with four chains of 10,000,000 replications, with sample frequency of 2,000 and discarding the first 25% of replicates as burn-in. Two independent runs were conducted to produce a consensus tree that we used to explore relationships between haplotypes. We considered the runs as having converged when their split frequency was <0.01 and the potential scale reduction factor was close to 1 [57]. The tree was rooted with *P. ornata* (Genbank AY221451.1). We also built a statistical parsimony network using TCS v1.7 [59]. Sites with missing data (nucleotides and gaps) were not considered when sequences were collapsed into haplotypes.

Neutrality tests (Tajima's D and F_S_) [60], [61] and pairwise mismatch distribution were used to detect any signal of recent demographic expansion or increase from a founder population. Geographical regions with negative neutrality indices and unimodal mismatch distributions are expected to show demographic expansion [62]. The raggedness statistic, r, was used to test whether the observed mismatch distribution was significantly different from the expected unimodal distribution [63]. All analyses and tests for significance were performed in DnaSP v5.10.01 with 10,000 coalescent simulations.

To compare the genetic diversity of *P. guimbeaui* with other species of the same genus, we compared π between homologous 464-bp *cyt b* sequences from *P. guimbeaui*, two subspecies of *Phelsuma astriata* (Seychelles small day gecko) and three subspecies of *Phelsuma sundbergi* (Seychelles giant day gecko), all of which are common in the Seychelles [64].

## Results

### Amplification success and genotyping errors

Amplification success of loci varied from 83.0 to 100.0% (mean ± SD 96.2±3.7%), except for *Pgu 043*, which amplified only 57.6% of the time and so was excluded from subsequent analyses (Table S3). Amplification success (proportion of loci amplifying) varied across samples from 61.7 to 100.0% (mean ± SD 93.7±6.1%). Sampled individuals with an amplification success of less than 70.0% were excluded from the analysis. Loci *Pgu 005* and *Pgu 033* were discarded because of stutter-band scoring errors. Three loci, *Pgu 009*, *Pgu 017* and *Pgu 040*, displayed minor evidence of genotyping errors, with a maximum error rate of 0.033 per allele (Table S3); these loci were retained because of the relatively low mean error rate of 0.004 for all 41 loci. After screening for data quality, a total of 260 geckos genotyped at 38 loci were retained for subsequent testing for HW disequilibrium, LD and the presence of null alleles.

### Selection of loci for population genetic analyses

Significant LD was observed in three pairs of loci. However, we retained these loci because the LD was not consistently high across all subpopulations. The number of loci in HW disequilibrium (Table S4) and significant null alleles (proportion >0.1) in the different subpopulations (Table S5) varied from zero to ten. Only loci showing evidence of null alleles and HW disequilibrium (P<0.05) in a maximum of three subpopulations were retained for analysis. Heterozygotes were present in males and females at every locus, indicating no linkage to sex chromosomes. We excluded loci with indications of selection, evidence of alleles differing by 1 base pair, and allele sizes greater than 500 base pairs because the ABI 3730 DNA analyser could not distinguish fragment sizes larger than 500 base pairs accurately. We retained 20 loci for the population genetics analyses (Table S3).

### Genetic diversity of subpopulations

The 20 retained markers were all highly polymorphic, with the number of alleles per locus ranging from 11 to 60 (Table S6). Genetic diversity was high at all sites: mean H_E_ varied from 0.844 to 0.891, H_O_ from 0.792 to 0.876, N_A_ from 10.6 to 15.5 and A_R_ from 8.4 to 10.7. P_A_ was observed in all the subpopulations and mean P_A_ varied from 0.400 to 0.960 (Table 1). Significant differences were detected in mean H_E_ (F_9_ = 9.12, P<0.001) and A_R_ (F_9_ = 10.17, P<0.001) between the following subpopulations: (i) subpopulation L10 had significantly lower H_E_ and A_R_ compared to all other subpopulations except L1 and L7; and (ii) L1 and L7 had significantly lower H_E_ and A_R_ compared to subpopulations L2, L3, L4, L5 and L6. There was also a significant positive F_IS_ varying between 0.006 and 0.108 in all subpopulations (Table 1), which indicated inbreeding [65].

**Table 1:** Population genetic indices (± standard deviation) for ten subpopulations of *Phelsuma guimbeaui*.

Subpop	A	H_E_	H_O_	N_A_	A_R_	P_A_	F_IS_
L1	22	0.852±0.018	0.818±0.019	10.7±3.1	8.7±2.2	0.400±0.727	0.042
L2	28	0.877±0.016	0.833±0.016	14.8±4.7	10.4±2.5	0.960±0.791	0.051
L3	29	0.880±0.014	0.817±0.016	13.9±4.8	10.0±2.6	0.800±1.060	0.074
L4	23	0.883±0.012	0.825±0.019	12.3±3.1	9.9±2.1	0.900±1.051	0.067
L5	29	0.889±0.013	0.795±0.017	14.5±.4	10.4±2.6	0.710±0.731	0.108
L6	29	0.891±0.014	0.853±0.015	15.5±5.4	10.7±2.8	0.830±0.894	0.043
L7	23	0.856±0.012	0.807±0.019	11.3±3.3	8.8±.2	0.500±0.611	0.059
L8	26	0.873±0.013	0.833±0.017	12.8±4.3	9.6±2.4	0.680±.964	0.047
L9	26	0.882±0.014	0.876±0.015	14.0±4.3	10.2±2.5	0.660±0.747	0.006
L10	25	0.844±0.016	0.792±0.019	10.6±.8	8.4±1.9	0.630±0.671	0.062

Subpop = subpopulation; A = number of individuals sampled; H_E_ = mean expected heterozygosity and standard deviation; H_O_ = mean observed heterozygosity and standard deviation; N_A_ = mean number of alleles and standard deviation; A_R_ = mean allelic diversity and standard deviation; P_A_ = mean private allelic richness and standard deviation; F_IS_ = inbreeding coefficient. N_A_, A_R_ and P_A_ were based on a minimum sample of 22 geckos per subpopulation.

Based on the maps of habitat loss (Figure 1), the different subpopulations have been isolated from each other for approximately 0 to 239 years (L2 and L6, 0 years; L4, 77 years; L7 and L10, 140 years; L1, L3, L8 and L9, 177 years; L5, 239 years). We found no correlation between genetic diversity and habitat loss measured as time of isolation (A_R_: r = −0.328, t_8_ = −0.981, P>0.05; H_E_: r = −0.183, t_8_ = −0.526, P>0.05).

### Population structure, differentiation and IBD

**Figure 3:**
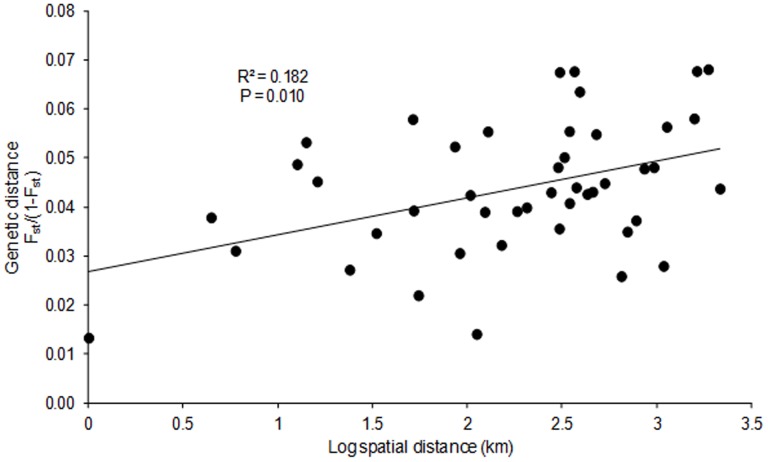
Isolation by distance (IBD) in *Phelsuma guimbeaui* using microsatellite markers. Genetic distance F_st_/(1-F_st_) is plotted against log spatial distance (km).

The high membership coefficients to genetic clusters (0.705 to 0.899) found using STRUCTURE supported strong genetic differentiation of subpopulations. The highest delta K and log probability values were observed at K = 9, suggesting that all subpopulations except L8 and L9 were genetically distinct (Figure 2a, b). On average, 86.6% (range 69.3 to 93.1%) of individuals were correctly assigned to their respective subpopulations using the assignment test in GENECLASS2 (Figure 2c). Using TESS, the lowest DIC was obtained for K = 10, indicating that all subpopulations were genetically distinct (Figure 2c). We found a significant pattern of IBD using the microsatellite genotypes (R^2^ = 0.182, P = 0.010) (Figure 3). Pairwise F_st_ comparisons among all subpopulations suggested that there was small to moderate genetic differentiation (P<0.001), with F_st_ estimates between 0.016 and 0.072 [66]. The greatest degrees of genetic differentiation were between subpopulations L1 and L2, L1 and L10, L1 and L8, L2 and L7, L2 and L10, L4 and L7, and L7 and L10. The lowest differentiation was found between subpopulations L2 and L6, and L8 and L9 (Table 2). Consistent with the analyses of population structure, the AMOVA confirmed the existence of significant genetic variation at different hierarchical levels, with 4% (F_9_ = 0.042, P<0.001) of variation occurring between subpopulations, 10% among individuals (F_250_ = 0.108, P<0.001) and 86% (F_260_ = 0.145, P<0.001) within individuals.

**Table 2:** Pairwise F_st_ values among the 10 subpopulations of *Phelsuma guimbeaui*; all values were significant at P<0.001 based on 999 permutations.

Subpop	L1	L2	L3	L4	L5	L6	L7	L8	L9	L10
**L1**										
**L2**	0.070									
**L3**	0.029	0.060								
**L4**	0.050	0.055	0.036							
**L5**	0.045	0.050	0.024	0.031						
**L6**	0.047	0.019	0.034	0.045	0.026					
**L7**	0.046	0.067	0.035	0.063	0.037	0.045				
**L8**	0.061	0.051	0.044	0.039	0.033	0.037	0.056			
**L9**	0.045	0.044	0.032	0.043	0.026	0.030	0.039	0.016		
**L10**	0.072	0.067	0.044	0.050	0.040	0.049	0.063	0.049	0.045	

Subpop = subpopulation.

### Bottlenecks, migration and effective population size

Using the mutation rate estimate for reptilian microsatellite loci [47], subpopulations L1, L3 and L5 showed evidence of recent bottlenecks, with a significant Wilcoxon's test (P<0.05) suggesting an excess of heterozygotes. However, no signs of a bottleneck were observed when the lower general vertebrate mutation rate was used [10]. Using the Bayesian approach in BAYESASS v1.3, the mean probability of no migration occurring per subpopulation in any generation was 0.833 (range 0.675 to 0.992) and the mean migration rate per generation was 0.019 (range 0.000 to 0.121). However, the estimated migration rates between subpopulations were different in each run and so the results were unreliable. No immigrants were detected in the STRUCTURE analysis (USEINFOPOP model). Because the migration results were inconsistent between the different methods, we used the first-generation migrants from GENECLASS2 to calculate the general migration rate [67] and found little evidence of migration across the ten subpopulations (Table S7). Modal estimates of N_e_ for the different subpopulations were small, ranging from 44 to 167 in N_e_ ESTIMATOR and 19 to 96 in MIGRATE (Table 3).

**Table 3:** Mean effective population sizes with 95% confidence intervals in parentheses for ten subpopulations of *Phelsuma guimbeaui* estimated using N_e_ ESTIMATOR and MIGRATE.

Subpopulation	N_e_ ESTIMATOR	MIGRATE
L1	123 (90–191)	19 (4–38)
L2	121 (100–154)	54 (33–77)
L3	145 (116–193)	57 (35–80)
L4	72 (60–91)	60 (34–88)
L5	125 (102–159)	96 (64–135)
L6	167 (133–224)	93 (51–125)
L7	106 (82–148)	26 (4–46)
L8	76 (64–92)	42 (19–71)
L9	121 (98–157)	75 (44–121)
L10	44 (39–51)	24 (6–43)

### Mitochondrial DNA

**Figure 4:**
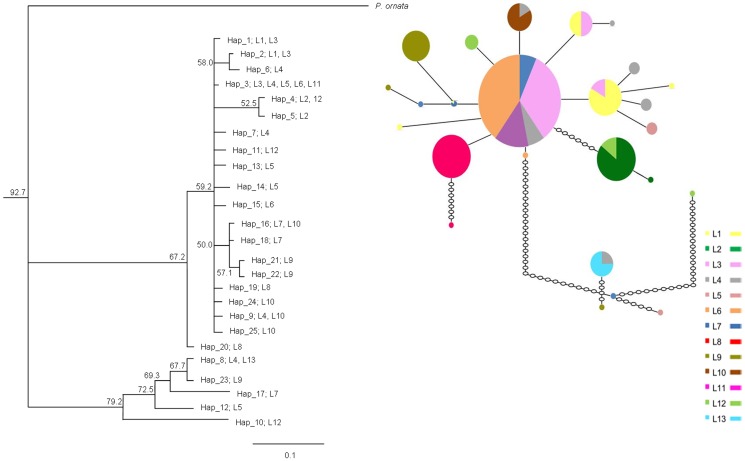
Phylogenetic relationships of mtDNA haplotypes in *Phelsuma guimbeaui*. The Bayesian tree was produced in MRBAYES with subpopulation identity (L1 to L13) shown at the end of each branch. In the parsimony network, the circles represent different haplotypes, with their size proportional to the number of geckos. Open circles represent predicted but missing or unsampled haplotypes.

We obtained partial *cyt b* with 464 base pairs (accession numbers HG779461-HG779540) and 1*6S rRNA* with 313 base pairs (accession numbers HG518676-HG518755). We analysed concatenated partial *cyt b* and 1*6S rRNA* sequences (777 base pairs) of 80 individuals from 13 subpopulations (Figure 2a, Table 4). No stop codon was identified, indicating that the true genes rather than nuclear pseudogenes were amplified. We identified 108 sites with missing data (unidentified nucleotides or gaps), 607 monomorphic sites and 62 polymorphic sites (ten singletons and 52 parsimony informative sites). Twenty-five unique haplotypes (Hap_1-25) were recorded in the parsimony network and phylogenetic tree (Figure 4). The number of haplotypes ranged from one to five per subpopulation, with Hap_3 (18.8%) being the most common (Figure 4, Table S8). Seven haplotypes were shared between different subpopulations: (i) Hap_1 and Hap_2 in L1 and L3; (ii) Hap_3 in L3, L4, L5, L6 and L11; (iii) Hap_4 in L2 and L12; (iv) Hap_8 in L4 and L13; (v) Hap_9 in L4 and L10; and (vi) Hap_16 in L7 and L10 (Figure 4, Table S8). Unique haplotypes were present in all subpopulations, except some of those where we had a very small sample size, i.e. L11 and L13, but not L12.

**Table 4:** Population statistics of the concatenated mtDNA sequences for 13 subpopulations of *Phelsuma guimbeaui*.

Subpop	Area	A	H_N_	Haplotype	H	π	D	Fs	Mismatch distribution	r
L1	0.002	7	2	Hap_1,2	0.476	0.001	0.687	1.741	Multimodal	0.042*
L2	0.200	7	2	Hap_4,5	0.286	<0.001	−1.021	−0.095	Multimodal	0.042*
L3	0.060	8	3	Hap_1,2,3	0.607	0.001	−0.084	0.044	Multimodal	0.042*
L4	0.050	6	5	Hap_3,6,7,8,9	0.933	0.022	−1.423	1.723	Multimodal	0.042
L5	0.010	8	4	Hap_3,12,13,14	0.857	0.027	0.558	6.934	Multimodal	0.042
L6	0.500	7	2	Hap_3,15	0.286	<0.001	−1.023	−0.095	Unimodal	0.042*
L7	0.010	7	3	Hap_16,17,18	0.524	0.013	−1.821*	5.811	Bimodal	0.042*
L8	0.030	8	2	Hap_19,20	0.250	0.003	−1.700*	4.213	Multimodal	0.042*
L9	0.004	7	3	Hap_21,22,23	0.714	0.017	−1.721*	4.221	Multimodal	0.042*
L10	0.004	8	3	Hap_9,16,24,25	0.643	0.002	−0.222	−0.470	Bimodal	0.042*
L11	0.500	1	-	Hap_3	-	-	-	-	-	-
L12	0.040	3	3	Hap_4,10,11	1.000	0.044	-	2.422	Multimodal	0.042
L13	0.003	3	1	Hap_8	0.667	0.002	-	1.133	Multimodal	0.042*
Overall		80	25		0.931	0.016	−0.856	−0.752	Multimodal	0.042

* denotes significant at P<0.05.

Subpop = subpopulation; Area = area of subpopulation in km^2^; A = number of individuals per subpopulation; H_N_ = number of haplotypes; H = haplotype diversity; π = nucleotide diversity; D = Tajima's D; Fs = Fu's Fs statistic; r = raggedness index.

Results for mismatch distribution, H and π per subpopulation and for the entire population, are shown in Table 4. Overall, the entire population had negative values for D and F_S_, but these were not significant, and a unimodal distribution with P>0.05 for the r index suggested demographic expansion. Bayesian and maximum-likelihood phylogenetic tree topologies were similar, and so only the Bayesian results are presented. The Bayesian topology shows clades with low support, with posterior probability <80% (Figure 4). *P. guimbeaui* was three to five times more genetically diverse than the Seychellois geckos, i.e. π = 0.015 in *P. guimbeaui* compared to π = 0.005 for *P. a. astriata*, π = 0.005 for *P. a. semicarinata*, π = 0.003 for *P. s. ladiguensis*, π = 0.004 for *P. s. longinsulae* and π = 0.003 for *P. s. sundbergi*.

### Extinction risks and retaining rare alleles through translocation

AlleleRetain simulations averaged across 1,000 replications over 50 years showed that the probability of survival for each subpopulation varied from 0.090 to 0.740, and the probability of retaining rare alleles varied from 0.025 to 0.292 when no migration occurred (Table 5). Under the different scenarios, the simulation results showed that an increase in the number of geckos included in the initial translocation will lead to an increase in the probability of survival and capture of rare alleles, but stabilised at around 20 individuals in most subpopulations (Table S1). With no assisted migration, the probabilities of survival and of retaining rare alleles were nearly zero, but increased significantly when assisted migrants were added to the new translocated population, with a maximum of 0.890 (CI 0.858–0.915) and 0.736 (CI 0.694–0.773) for the probability of survival and retention of rare alleles, respectively (Table S1). The maximum probabilities of survival and of rare allele retention were highest when assisted migration occurred yearly after initial translocation, but gradually decreased to a minimum of 0.206 (CI 0.171–0.244) for probability of survival and 0.126 (CI 0.098–0.159) for retention of rare alleles when assisted migration was only conducted every five years (Table S1).

**Table 5:** Simulation showing the probability of survival and retaining rare alleles, with 95% confidence intervals in parentheses, over 50 years in ten subpopulations of *Phelsuma guimbeaui* in the absence of migration.

Subpopulation	Estimated population size	Probability of survival	Probability of retaining rare alleles
L1	1230	0.706 (0.663–0.745)	0.276 (0.238–0.318)
L2	1210	0.740 (0.711–0.767)	0.292 (0.264–0.321)
L3	1450	0.540 (0.509–0.571)	0.191 (0.167–0.217)
L4	720	0.217 (0.192–0.244)	0.046 (0.034–0.061)
L5	1250	0.443 (0.412–0.474)	0.124 (0.105–0.146)
L6	1670	0.633 (0.602–0.663)	0.243 (0.217–0.271)
L7	1060	0.380 (0.350–0.411)	0.116 (0.097–0.138)
L8	760	0.243 (0.217–0.271)	0.084 (0.068–0.103)
L9	1210	0.438 (0.407–0.469)	0.130 (0.110–0.153)
L10	440	0.090 (0.073–0.110)	0.025 (0.017–0.037)

The estimated population size (N_e_ ×10) with a default rare allele frequency of 0.05 was implemented into the starting parameters of all models.

## Discussion

The microsatellite analysis revealed a high degree of population structure and genetic diversity across ten isolated subpopulations. Similarly, the mtDNA data revealed high levels of genetic diversity across the fragmented population despite severe habitat loss and isolation during the last 250 years. Phylogenetic analyses based on mtDNA suggest that the 13 subpopulations were all formerly part of a panmictic population, while the microsatellite analyses indicate that the subpopulations became genetically differentiated through habitat loss and isolation following human colonisation of Mauritius. The N_e_ was low and simulations suggested that there is a high risk of genetic erosion and extinctions in the next 50 years if subpopulations remain in isolation. While our analyses are based on microsatellite data from ten, and mtDNA data from 13, of the 30 known subpopulations of *P. guimbeaui*, we believe that our data are representative as we covered the known range of *P. guimbeaui* and the non-sampled subpopulations were scattered among those that we did sample.

### Population structure, genetic diversity and migration

Since this is the first study to use nuclear markers to examine genetic diversity in the genus *Phelsuma*, comparisons with congeneric species were not possible. The microsatellite measures of genetic diversity were relatively high compared to other reptiles [68], [69] despite subpopulations having been isolated for periods up to 239 years. However, we could not find any evidence for a correlation between genetic diversity and time of isolation. Similarly, the high level of nucleotide diversity in *P. guimbeaui* compared to common species of *Phelsuma* in the Seychelles suggests that the various subpopulations of *P. guimbeaui* are still genetically diverse. The negative D and F_S_, while not significant, and their unimodal distributions imply that *P. guimbeaui* has experienced a recent population expansion. This could be due to an unexpectedly high proportion of rare alleles originating from a founder effect [70], possibly because *P. guimbeaui* has colonised expanding habitats dominated by *Eucalyptus tereticornis*, *Haematoxylum campechianum*, *Mangifera indica* and *Terminalia arjuna*. All the subpopulations we sampled, except L2, L6, L11 and L12, were in exotic plantations. However, since these plantations are small and widely scattered, natural migration between them is unlikely and they do not provide suitable alternative habitats for the long-term conservation of *P. guimbeaui*.

Our analyses suggest that either nine (STRUCTURE) or all ten (TESS and GENECLASS2) of the sampled subpopulations were genetically distinct. There was a relatively moderate or high level of admixture between subpopulations L2 and L6, and between L8 and L9, which may be due to the relative proximity of these paired sites or connectivity. While most sites were separated by large expanses of urbanisation or agriculture, subpopulations L2 and L6 were in a continuous forest within the Black River mountains but isolated by forest dominated by invasive plants, predominantly *Psidium cattleianum*. However, individual *P. guimbeaui* were seen on scattered native trees in areas of invaded forest, suggesting that low levels of migration may be occurring. While subpopulations L8 and L9 were separated by 0.6 km of unsuitable habitat, including a 70 m wide strip of bare land, the admixture results suggest that migration could still be occurring between them, even though the other analyses tend to suggest little or no migration. AMOVA and F_st_ analyses identified genetic structure with small to moderate levels of genetic differentiation between the geographically isolated subpopulations. A small F_st_ can be a sign of historical differentiation and recent gene flow [71] or a signal of shared descent [72]. The relatively low F_st_ values between subpopulations L2 and L6 and L8 and L9 may also be due to the high level of admixture between these pairs of subpopulations, even though TESS and GENECLASS2 predicted that they were genetically different. These results suggest that habitat fragmentation (or lack of connectivity) has had an impact on genetic differentiation in many subpopulations by limiting migration. There are two plausible non-mutually exclusive explanations that may account for the level of genetic differentiation observed among the subpopulations. First, restricted dispersal can produce a pattern of IBD [73]. According to this model, gene flow will decline linearly with geographical distance. Second, isolation due to barriers roads and agricultural areas may have enhanced genetic differentiation. More than 95% of Mauritius' native forest has been lost since human colonisation in 1638 [74]. Habitat destruction coincided with planting of the four types of exotic plantation that provide alternative habitats for *P. guimbeaui*. The initial founder effect of these colonisations, together with the cumulative effects of genetic drift and low effective population size, may have promoted genetic differentiation.

### Effective population size and bottlenecks

Values for N_e_ ESTIMATOR were consistently higher than those obtained with MIGRATE, and the former LD-based method may provide a better estimate of N_e_ because it records recent N_e_ (up to two generations) and so is less likely to be affected by historical events [75]. We consider our N_e_ estimates to be small and so these subpopulations are at high risk of extinction [76]. We had expected that many subpopulations would have been subjected to a genetic bottleneck. However, only subpopulations L1, L3 and L5 showed evidence of a recent bottleneck when the reptilian multiple-step mutation rate was used for demographic models. This could be because the reptilian mutation rate was inappropriate, since it was estimated from just one species of skink [47]. Additionally, this method will only detect recent bottlenecks and is sensitive to small sample sizes [10], and the effects of gene flow [77], so our bottleneck results for *P. guimbeaui* should be interpreted with caution.

### Phylogenetic relationships of haplotypes

Phylogenetic analyses provided strong evidence that the spatial genetic structure and differentiation were absent in *P. guimbeaui* prior to habitat loss. Shared haplotypes among the subpopulations suggest that they originally formed part of a panmictic population. For example, Hap_3 was shared by five subpopulations, among which L3 and L11 were more than 20 km apart. The absence of genetic structure within the mtDNA data is also supported by historical vegetation maps, which show that most of the western part of Mauritius had continuous forest cover, and *P. guimbeaui* may have occurred throughout these lowland dry forests prior to human colonisation. This also suggests that *P. guimbeaui* was widely dispersed when the forest was not fragmented.

Contrasting signals of genetic structure between nuclear and mtDNA data have been observed in other species [78]. There are several alternative explanations, including differences in mutation rate and sex-biased dispersal. MtDNA has a lower mutation rate than microsatellites [47], [79] and mtDNA lineages may not yet have become differentiated following the onset of habitat fragmentation [7]. The population structure revealed by the microsatellite genotypes is consistent with the extent of landscape fragmentation. Most forest fragments containing subpopulations have been isolated from each other within the last two centuries, suggesting that the population structure occurred as a consequence of recent habitat loss. Alternatively, gene flow may be promoted solely by females, since males are more immobile [78]. However, we consider this is an unlikely explanation of the lack of genetic structure at mtDNA markers because of the low dispersal rates in both sexes of *P. guimbeaui* (S. Buckland, unpublished data) and the observed strong differentiation of nuclear markers.

### Implications for conservation

Our genetic data suggest that *P. guimbeaui* was a panmictic population until relatively recently, which has become differentiated through anthropogenic habitat loss and isolation. There is, therefore, no reason to maintain the genetic identity of individual subpopulations and little risk of outbreeding depression should subpopulations be mixed. Conservation management is a priority, since there is a high risk of genetic erosion and extinctions in the next 50 years, even without further habitat loss and other anthropogenic changes. Possible interventions based on our genetic data are: (i) the construction of habitat corridors linking closely-neighbouring subpopulations; (ii) the restoration and expansion of the native habitats in and around existing subpopulations; and (iii) the translocation of individuals to enhance the genetic diversity of viable subpopulations and/or establish one or more new populations.

Continuing habitat loss in Mauritius suggests that some subpopulations are likely to be lost in the next decade: we sampled nine such subpopulations. Given the immediacy of the threat, and that the construction of habitat corridors linking some of them and/or habitat restoration are not realistic options, the only practical solution is to translocate individuals from these nine subpopulations to reinforce viable subpopulations, if there is habitat to support additional geckos, or move them to suitable habitat patches to establish new population(s) [80]. We used AlleleRetain to see which management option would maximise the retention of allelic diversity in any new population that might be established by translocation. This program is designed to examine options when potential new populations are of limited size and cannot be supported by natural immigration [50]. To capture more than 80% of the rare alleles in each subpopulation, initially a minimum of 20 adult geckos would need to be translocated and established from each subpopulation, with assisted migration of 10 geckos each year thereafter. All calculations were based on single subpopulations and it was assumed that each subpopulation would be translocated to a different site. Mixing genetically different subpopulations would increase the proportion of rare alleles and reduce the number of geckos that would need to be translocated.

However, translocation of a selected number of animals is only practical if the host subpopulation is viable in the medium to longer term. Since these nine subpopulations (L1, L3, L4, L5, L7, L8, L9, L10, L13) are highly threatened by further habitat loss, it is unlikely that any will persist for more than a decade. So it may be more logical to translocate as many geckos as possible from each subpopulation in one operation, even if it hastens the ultimate loss of that subpopulation. Nine other subpopulations not sampled during this study (Figure 1) are equally threatened by habitat loss and so the same rationale applies. Data from the subpopulations we sampled strongly suggest that the nine subpopulations we did not sample will also be genetically different as they are small and completely isolated. So their translocation should enhance the overall genetic diversity preserved by conservation action.

Only four of the subpopulations we examined (L2, L6, L11 and L12) were still viable, and they were all within the Black River mountains, the largest area of remnant native forest in Mauritius. There were eight other subpopulations within the Black River mountains, which we did not sample but were probably also viable. Habitat restoration and expansion within this mountain range would support the long-term management of the species and enable *P. guimbeaui* to expand its range in the Black River mountains. The most practical solution would be to create habitat corridors linking those areas of native forest that still harbour relatively large subpopulations of *P. guimbeaui*. While this will increase migration between the 12 viable subpopulations and decrease intra-subpopulation inbreeding, this is a long-term strategy and so assisted migration within the Black River mountains should be considered as an interim option. However, prior to any such intervention, data are needed to confirm the viability and breeding success of translocated geckos following release into a new population. Post-release data are also needed to assess whether greater genetic diversity is retained by releasing geckos “rescued” from non-viable subpopulations into a viable subpopulation or using them to establish a new population.

## Conclusions

Although the immediate threat facing *P. guimbeaui* is habitat destruction, our data highlight the importance of genetic studies in guiding conservation management. Maximising the retention of genetic diversity is important and we identified two conservation measures to maximise the retention of genetic diversity in *P. guimbeaui*. We recommend: (i) a short-term rescue action by translocating as many geckos as possible from the 18 subpopulations threatened by imminent habitat loss; and (ii) a long-term action to restore habitats in the Black River mountains to link the 12 potentially viable subpopulations by habitat corridors. Since habitat management is a long-term strategy, especially since *P. guimbeaui* is a habitat specialist relying on high native tree diversity and large tall trees with numerous cavities, assisted migration among the 12 viable subpopulations should reduce the risks of genetic degradation in the short term. However, data are needed on the survival and breeding success of translocated geckos used to reinforce viable subpopulations or to establish new populations prior to the implementing any program of assisted migration.

## Supporting Information

Table S1 Simulation results from R package AlleleRetain. The table shows the probability of survival and genetic diversity after 50 years (1000 replications) in different scenarios in each subpopulation (Subpop) of *Phelsuma guimbeaui*: (i) initial number of translocated individuals (StartN) varied from 10–40; (ii) number of assisted migrants after translocation (MigrN) ranged from 0–30; and (iii) frequency at which assisted migrants were translocated (Frequency) confined from one to five years. R and simulations codes are given below the table.
(DOC)

Table S2 Mitochondrial DNA primers in *Phelsuma guimbeaui*.
(DOC)

Table S3 Screening for data quality to select the best loci for analyses of molecular variation in *Phelsuma guimbeaui*.
(DOC)

Table S4 The number of subpopulations of *Phelsuma guimbeaui* not in Hardy-Weinberg equilibrium (P<0.05) at the different loci.
(DOC)

Table S5 Null allele frequencies in ten subpopulations of *Phelsuma guimbeaui*.
(DOC)

Table S6 Number of alleles observed for the 20 loci used in the microsatellite analyses of *Phelsuma guimbeaui*.
(DOC)

Table S7 The mean probability of no migration and migration among ten subpopulations of *Phelsuma guimbeaui* obtained in GENECLASS2.
(DOC)

Table S8 Haplotype distribution and frequency in 80 individuals from 13 subpopulations of *Phelsuma guimbeaui*.
(DOC)

## References

[1] Frankham R, Ballou JD, Briscoe DA (2010) Introduction to conservation genetics. Cambridge: Cambridge University Press. 643 p.

[2] Janečka JE, Tewes ME, Laack LL, Grassman LI, Haines AM, et al (2008) Small effective population sizes of two remnant ocelot populations (*Leopardus pardalis albescens*) in the United States. Conservation Genetics 9: 869–878 10.1007/s10592-007-9412-1

[3] Slate J, Kruuk LEB, Marshall TC, Pemberton JM, Clutton-Brock TH (2000) Inbreeding depression influences lifetime breeding success in a wild population of red deer (*Cervus elaphus*). Proceedings of the Royal Society of London B 267: 1657–1662 10.1098/rspb.2000.1192 PMC1690715 11467429

[4] Újvári B, Madsen T, Kotenko T, Olsson M, Shine R, et al (2002) Low genetic diversity threatens imminent extinction for the Hungarian meadow viper (*Vipera ursinii rakosiensis*). Biological Conservation 105: 127–130 10.1016/S0006-3207(01)00176-8

[5] Liberg O, Andrén H, Pedersen H-C, Sand H, Sejberg D, et al (2005) Severe inbreeding depression in a wild wolf (*Canis lupus*) population. Biology Letters 1: 17–20 10.1098/rsbl.2004.0266 17148117 PMC1629062

[6] Bijlsma R, Bundgaard J, Boerema AC (2000) Does inbreeding affect the extinction risk of small populations?: predictions from *Drosophila* . Journal of Evolutionary Biology 13: 502–514 10.1046/j.1420-9101.2000.00177.x

[7] Dixo M, Metzger JP, Morgante JS, Zamudio KR (2009) Habitat fragmentation reduces genetic diversity and connectivity among toad populations in the Brazilian Atlantic Coastal Forest. Biological Conservation 142: 1560–1569 10.1016/j.biocon.2008.11.016

[8] Schtickzelle N, Mennechez G, Baguette M (2006) Dispersal depression with habitat fragmentation in the bog fritillary butterfly. Ecology 87: 1057–1065 10.1890/0012-9658 16676549

[9] Da Silva A, Luikart G, Yoccoz NG, Cohas A, Allainé D (2006) Genetic diversity-fitness correlation revealed by microsatellite analyses in European alpine marmots (*Marmota marmota*). Conservation Genetics 7: 371–382 10.1007/s10592-005-9048-y

[10] Peery MZ, Kirby R, Reid BN, Stoelting R, Doucet-Bëer E, et al (2012) Reliability of genetic bottleneck tests for detecting recent population declines. Molecular Ecology 21: 3403–3418 10.1111/j.1365-294X.2012.05635.x 22646281

[11] Cheke A, Hume J (2008) Lost land of the dodo: an ecological history of Mauritius, Réunion & Rodrigues. London: Poyser. 464 p.

[12] Arnold EN (2000) Using fossils and phylogenies to understand evolution of reptile communities on islands. Isolated vertebrate communities in the tropics. Bonner Zoologische Monographien 46: 309–323.

[13] Buckland S, Horsburgh GJ, Dawson DA, Cole NC, Krupa AP, et al (2013) Isolation and characterisation of Mauritius lowland day gecko *Phelsuma guimbeaui* microsatellite loci. Conservation Genetics Resources 5: 1013–1018 10.1007/s12686-013-9957-x

[14] Nicholls JA, Double MC, Rowell DM, Magrath RD (2000) The evolution of cooperative and pair breeding in thornbills *Acanthiza* (Pardalotidae). Journal of Avian Biology 31: 165–176 10.1034/j.1600-048X.2000.310208.x

[15] Kenta T, Gratten J, Haigh NS, Hinten GN, Slate J, et al (2008) Multiplex SNP-SCALE: a cost-effective medium-throughput single nucleotide polymorphism genotyping method. Molecular Ecology Resources 8: 1230–1238 10.1111/j.1755-0998.2008.02190.x 21586010

[16] Hoffman JI, Amos W (2005) Microsatellite genotyping errors: detection approaches, common sources and consequences for paternal exclusion. Molecular Ecology 14: 599–612 10.1111/j.1365-294X.2004.02419.x 15660949

[17] Park SDE (2002) Trypanotolerance in West African cattle and the population genetic effects of selection. University of Dublin: PhD thesis. 241 p.

[18] Van Oosterhout C, Hutchinson WF, Wills DPM, Shipley P (2004) MICRO-CHECKER: software for identifying and correcting genotyping errors in microsatellite data. Molecular Ecology Notes 4: 535–538 10.1111/j.1471-8286.2004.00684.x

[19] Kalinowski ST, Wagner AP, Taper ML (2006) ML-RELATE: a computer program for maximum likelihood estimation of relatedness and relationship. Molecular Ecology Notes 6: 576–579 10.1111/j.1471-8286.2006.01256.x

[20] Anderson EC, Dunham KK (2008) The influence of family groups on inferences made with the program Structure. Molecular Ecology Resources 8: 1219–1229 10.1111/j.1755-0998.2008.02355.x 21586009

[21] Antao T, Lopes A, Lopes RJ, Beja-Pereira A, Luikart G (2008) LOSITAN: a workbench to detect molecular adaptation based on a *F_st_*-outlier method. BMC Bioinformatics 9 323: 10.1186/1471-2105-9-323 PMC2515854 18662398

[22] Raymond M, Rousset F (1995) GENEPOP (version 1.2): population genetics software for exact tests and ecumenicism. Journal of Heredity 86: 248–249 doi: cgi/content/short/86/3/248

[23] Verhoeven KJF, Simonsen KL, McIntyre LM (2005) Implementing false discovery rate control: increasing your power. Oikos 108: 643–647 10.1111/j.0030-1299.2005.13727.x

[24] Kalinowski ST, Taper ML, Marshall TC (2007) Revising how the computer program CERVUS accommodates genotyping error increases success in paternity assignment. Molecular Ecology 16: 1099–1106 10.1111/j.1365-294X.2007.03089.x 17305863

[25] Kalinowski ST (2005) HP-RARE 1.0: a computer program for performing rarefaction on measures of allelic richness. Molecular Ecology Notes 5: 187–189 10.1111/j.1471-8286.2004.00845.x

[26] Pritchard JK, Stephens M, Donnelly P (2000) Inference of population structure using multilocus genotype data. Genetics 155: 945–959. 10835412 10.1093/genetics/155.2.945 PMC1461096

[27] Falush D, Stephens M, Pritchard JK (2003) Inference of population structure using multilocus genotype data: linked loci and correlated allele frequencies. Genetics 164: 1567–1587. 12930761 10.1093/genetics/164.4.1567 PMC1462648

[28] Evanno G, Regnaut S, Goudet J (2005) Detecting the number of clusters of individuals using the software STRUCTURE: a simulation study. Molecular Ecology 14: 2611–2620 10.1111/j.1365-294X.2005.02553.x 15969739

[29] Earl DA, von Holdt BM (2012) STRUCTURE HARVESTER: a website and program for visualizing STRUCTURE output and implementing the Evanno method. Conservation Genetics Resources 4: 359–361 10.1007/s12686-011-9548-7

[30] Jakobsson M, Rosenberg NA (2007) CLUMPP: a cluster matching and permutation program for dealing with label switching and multimodality in analysis of population structure. Bioinformatics 23: 1801–1806 10.1093/bioinformatics/btm233 17485429

[31] Rosenberg NA (2004) DISTRUCT: a program for the graphical display of population structure. Molecular Ecology Notes 4: 137–138 10.1046/j.1471-8286.2003.00566.x

[32] Chen C, Durand E, Forbes F, Francois O (2007) Bayesian clustering algorithms ascertaining spatial population structure: a new computer program and a comparison study. Molecular Ecology Notes 7: 747–756 10.1111/j.1471-8286.2007.01769.x

[33] Piry S, Alapetite A, Cornuet J-M, Paetkau D, Baudouin L, et al (2004) GENECLASS2: a software for genetic assignment and first-generation migrant detection. Journal of Heredity 95: 536–539 10.1093/jhered/esh074 15475402

[34] Rannala B, Mountain JL (1997) Detecting immigration by using multilocus genotypes. Proceedings of the National Academy of Sciences of the USA 94: 9197–9201. 9256459 10.1073/pnas.94.17.9197 PMC23111

[35] Paetkau D, Slade R, Burden M, Estoup A (2004) Genetic assignment methods for the direct, real-time estimation of migration rate: a simulation-based exploration of accuracy and power. Molecular Ecology 13: 55–65 10.1046/j.1365-294X.2003.02008.x 14653788

[36] Weir BS, Cockerham CC (1984) Estimating *F*-statistics for the analysis of population structure. Evolution 38: 1358–1370 10.2307/2408641 28563791

[37] Peakall R, Smouse PE (2012) GenAlEx 6.5: genetic analysis in Excel. Population genetic software for teaching and research - an update. Bioinformatics 28: 2537–2539 10.1093/bioinformatics/bts460 22820204 PMC3463245

[38] Excoffier L, Smouse PE, Quattro JM (1992) Analysis of molecular variance inferred from metric distances among DNA haplotypes: application to human mitochondrial DNA restriction data. Genetics 131: 479–491. 1644282 10.1093/genetics/131.2.479 PMC1205020

[39] Hardy OJ, Vekemans X (2002) SPAGeDi: a versatile computer program to analyse spatial genetic structure at the individual or population levels. Molecular Ecology Notes 2: 618–620 10.1046/j.1471-8278.2002.00305.x

[40] Wright S (1943) Isolation by distance. Genetics 28: 114–138. 17247074 10.1093/genetics/28.2.114 PMC1209196

[41] Vaughan RE, Wiehe PO (1937) Studies on the vegetation of Mauritius: I. A preliminary survey of the plant communities. Journal of Ecology 25: 289–343.

[42] Page W, D'Argent G (1997) A vegetation survey of Mauritius. Unpublished report commissioned by IUCN, BASEL. Port Louis: Mauritian Wildlife Foundation. 111 p.

[43] Nyhagen DF, Kragelund C, Olesen JM, Jones CG (2001) Insular interactions between lizards and flowers: flower visitation by an endemic Mauritian gecko. Journal of Tropical Ecology 17: 755–761.

[44] Ovenden JR, Peel D, Street R, Courtney AJ, Hoyle SD, et al (2007) The genetic effective and adult census size of an Australian population of tiger prawns (*Penaeus esculentus*). Molecular Ecology 16: 127–138 10.1111/j.1365-294X.2006.03132.x 17181726

[45] Beerli P (2006) Comparison of Bayesian and maximum-likelihood inference of population genetic parameters. Bioinformatics 22: 341–345 10.1093/bioinformatics/bti803 16317072

[46] Cornuet JM, Luikart G (1996) Description and power analysis of two tests for detecting recent population bottlenecks from allele frequency data. Genetics 144: 2001–2014. 8978083 10.1093/genetics/144.4.2001 PMC1207747

[47] Gardner MG, Bull CM, Cooper SJB, Duffield GA (2000) Microsatellite mutations in litters of the Australian lizard *Egernia stokesii* . Journal of Evolutionary Biology 13: 551–560 10.1046/j.1420-9101.2000.00189.x

[48] Wilson GA, Rannala B (2003) Bayesian inference of recent migration rates using multilocus genotypes. Genetics 163: 1177–1191. 12663554 10.1093/genetics/163.3.1177 PMC1462502

[49] Pritchard JK, Wen X, Falush D (2009) Documentation for *structure* software: Version 2.3. http://kinglab.eeb.lsa.umich.edu/EEID/eeid/evolution/Popgen_EEID_2012/Manuals/STRUCTURE_Manual.pdf.

[50] Weiser EL, Grueber CE, Jamieson IG (2012) AlleleRetain: a program to assess management options for conserving allelic diversity in small, isolated populations. Molecular Ecology Resources 12: 1161–1167 10.1111/j.1755-0998.2012.03176.x 22925629

[51] Palstra FP, Fraser DJ (2012) Effective/census population size ratio estimation: a compendium and appraisal. Ecology and Evolution 2: 2357–2365 10.1002/ece3.329 23139893 PMC3488685

[52] Austin JJ, Arnold EN, Jones CG (2004) Reconstructing an island radiation using ancient and recent DNA: the extinct and living day geckos (*Phelsuma*) of the Mascarene islands. Molecular Phylogenetics and Evolution 31: 109–122 10.1016/j.ympev.2003.07.011 15019612

[53] Rozen S, Skaletsky H (2000) Primer3 on the WWW for general users and for biologist programmers. In: Misener S, Krawetz SA, eds. Methods in molecular biology, vol. 132: bioinformatics methods and protocols. Totowa, NJ: Humana Press. pp 365–386. 10.1385/1-59259-192-2:365 10547847

[54] Kumar S, Nei M, Dudley J, Tamura K (2008) MEGA: a biologist-centric software for evolutionary analysis of DNA and protein sequences. Briefings in Bioinformatics 9: 299–306 10.1093/bib/bbn017 18417537 PMC2562624

[55] Nei M (1987) Molecular evolutionary genetics. New York: Columbia University Press. 526 p.

[56] Rozas J, Sánchez-DelBarrio JC, Messeguer X, Rozas R (2003) DnaSP, DNA polymorphism analyses by the coalescent and other methods. Bioinformatics 19: 2496–2497 10.1093/bioinformatics/btg359 14668244

[57] Huelsenbeck JP, Ronquist F (2001) MRBAYES: Bayesian inference of phylogenetic trees. Bioinformatics 17: 754–755 10.1093/bioinformatics/17.8.754 11524383

[58] Posada D, Crandall KA (1998) MODELTEST: testing the model of DNA substitution. Bioinformatics 14: 817–818 10.1093/bioinformatics/14.9.817 9918953

[59] Clement M, Posada D, Crandall KA (2000) TCS: a computer program to estimate gene genealogies. Molecular Ecology 9: 1657–1659. 11050560 10.1046/j.1365-294x.2000.01020.x

[60] Tajima F (1989) Statistical method for testing the neutral mutation hypothesis by DNA polymorphism. Genetics 123: 585–595. 2513255 10.1093/genetics/123.3.585 PMC1203831

[61] Fu Y-X (1997) Statistical tests of neutrality of mutations against population growth, hitchhiking and background selection. Genetics 147: 915–925. 9335623 10.1093/genetics/147.2.915 PMC1208208

[62] Slatkin M, Hudson RR (1991) Pairwise comparisons of mitochondrial DNA sequences in stable and exponentially growing populations. Genetics 129: 555–562. 1743491 10.1093/genetics/129.2.555 PMC1204643

[63] Rogers AR, Harpending H (1992) Population growth makes waves in the distribution of pairwise genetic differences. Molecular Biology and Evolution 9: 552–569. 1316531 10.1093/oxfordjournals.molbev.a040727

[64] Rocha S, Posada D, Harris DJ (2013) Phylogeography and diversification history of the day-gecko genus *Phelsuma* in the Seychelles islands. BMC Evolutionary Biology 13 3: 10.1186/1471-2148-13-3 PMC3598968 23289814

[65] Keller LF, Waller DM (2002) Inbreeding effects in wild populations. Trends in Ecology & Evolution 17: 230–241 10.1016/S0169-5347(02)02489-8

[66] Hartl DL, Clark AG (1997) Principles of population genetics. Sunderland: Sinauer. 542 p.

[67] Manel S, Gaggiotti OE, Waples RS (2005) Assignment methods: matching biological questions with appropriate techniques. Trends in Ecology & Evolution 20: 136–142 10.1016/j.tree.2004.12.004 16701357

[68] Levy E, Kennington WJ, Tomkins JL, LeBas NR (2012) Phylogeography and population genetic structure of the ornate dragon lizard, *Ctenophorus ornatus* . PLoS One 7 10: e46351 10.1371/journal.pone.0046351 23049697 PMC3462208

[69] Balmer O, Ciofi C, Galbraith DA, Swingland IR, Zug GR, et al (2011) Population genetic structure of Aldabra giant tortoises. Journal of Heredity 102: 29–37 10.1093/jhered/esq096 20805288

[70] Wang J-P, Lin H-D, Huang S, Pan C-H, Chen X-L, et al (2004) Phylogeography of *Varicorhinus barbatulus* (Cyprinidae) in Taiwan based on nucleotide variation of mtDNA and allozymes. Molecular Phylogenetics and Evolution 31: 1143–1156 10.1016/j.ympev.2003.10.001 15120406

[71] Landguth EL, Cushman SA, Schwartz MK, McKelvey KS, Murphy M, et al (2010) Quantifying the lag time to detect barriers in landscape genetics. Molecular Ecology 19: 4179–4191 10.1111/j.1365-294X.2010.04808.x 20819159

[72] Wright S (1969) Evolution and the genetics of populations, Volume 2: The theory of gene frequencies. Chicago: University Chicago Press. 520 p.

[73] Meirmans PG (2012) The trouble with isolation by distance. Molecular Ecology 21: 2839–2846 10.1111/j.1365-294X.2012.05578.x 22574758

[74] Safford RJ (1997) A survey of the occurrence of native vegetation remnants on Mauritius in 1993. Biological Conservation 80: 181–188 10.1016/S0006-3207(96)00048-1

[75] Palsbøll PJ, Peery MZ, Olsen MT, Beissinger SR, Bérubé M (2013) Inferring recent historic abundance from current genetic diversity. Molecular Ecology 22: 22–40 10.1111/mec.12094 23181682

[76] Franklin IR, Frankham R (1998) How large must populations be to retain evolutionary potential? Animal Conservation 1: 69–70 10.1111/j.1469-1795.1998.tb00228.x.

[77] Busch JD, Waser PM, DeWoody JA (2007) Recent demographic bottlenecks are not accompanied by a genetic signature in banner-tailed kangaroo rats (*Dipodomys spectabilis*). Molecular Ecology 16: 2450–2462 10.1111/j.1365-294X.2007.03283.x 17561905

[78] Küpper C, Edwards SV, Kosztolányi A, Alrashidi M, Burke T, et al (2012) High gene flow on a continental scale in the polyandrous Kentish plover *Charadrius alexandrinus* . Molecular Ecology 21: 5864–5879 10.1111/mec.12064 23094674

[79] Brunner PC, Douglas MR, Bernatchez L (1998) Microsatellite and mitochondrial DNA assessment of population structure and stocking effects in Arctic charr *Salvelinus alpinus* (Teleostei: Salmonidae) from central Alpine lakes. Molecular Ecology 7: 209–223 10.1046/j.1365-294x.1998.00341.x

[80] Seddon PJ (2010) From reintroduction to assisted colonization: moving along the conservation translocation spectrum. Restoration Ecology 18: 796–802 10.1111/j.1526-100X.2010.00724.x

