# Supplementary material for: High Risks of Losing Genetic Diversity in an Endemic Mauritian Gecko: Implications for Conservation
Source: PLoS One. 2014 Jun 25;9(6):e93387. doi: 10.1371/journal.pone.0093387 (PMC4070904; doi:10.1371/journal.pone.0093387)
Supplement: Table S1 — Simulation results from R package AlleleRetain. The table shows the probability of survival and genetic diversity after 50 years (1000 replications) in different scenarios in each subpopulation (Subpop) of Phelsuma guimbeaui: (i) initial number of translocated individuals (StartN) varied from 10–40; (ii) number of assisted migrants after translocation (MigrN) ranged from 0–30; and (iii) frequency at which assisted migrants were translocated (Frequency) confined from one to five years. R and simulations codes are given below the table. (DOC) [file pone.0093387.s001.doc]

**Table S1.** Simulation results from R package AlleleRetain. The table shows the probability of survival and genetic diversity after 50 years (1000 replications) in different scenarios in each subpopulation (Subpop) of *Phelsuma guimbeaui*: (i) initial number of translocated individuals (StartN) varied from 10-40; (ii) number of assisted migrants after translocation (MigrN) ranged from 0-30; and (iii) frequency at which assisted migrants were translocated (Frequency) confined from one to five years. R and simulations codes are given below the table.

| **Subpop** | **StartN** | **MigrN** | **Frequency** | **Probability of survival (95% confidence interval)** | **Genetic diversity retained (95% confidence interval)** |
| --- | --- | --- | --- | --- | --- |
| L1 | 10 | 10 | 3 | 0.458 (0.413 - 0.502) | 0.214 (0.179 - 0.253) |
| L1 | 20 | 10 | 3 | 0.632 (0.587 - 0.674) | 0.312 (0.271 - 0.354) |
| L1 | 30 | 10 | 3 | 0.616 (0.571 - 0.658) | 0.312 (0.271 - 0.354) |
| L1 | 40 | 10 | 3 | 0.688 (0.645 - 0.728) | 0.338 (0.296 - 0.381) |
| L1 | 10 | 0 | 3 | 0.014 (0.006 - 0.029) | 0.000 (0.000 - 0.009) |
| L1 | 10 | 20 | 3 | 0.854 (0.819 - 0.883) | 0.608 (0.563 - 0.650) |
| L1 | 10 | 30 | 3 | 0.850 (0.814 - 0.879) | 0.698 (0.655 - 0.737) |
| L1 | 10 | 10 | 1 | 0.850 (0.814 - 0.879) | 0.698 (0.655 - 0.737) |
| L1 | 10 | 10 | 2 | 0.680 (0.636 - 0.720) | 0.506 (0.461 - 0.550) |
| L1 | 10 | 10 | 3 | 0.410 (0.366 - 0.454) | 0.192 (0.158 - 0.229) |
| L1 | 10 | 10 | 4 | 0.282 (0.243 - 0.324) | 0.138 (0.109 - 0.172) |
| L1 | 10 | 10 | 5 | 0.210 (0.175 - 0.248) | 0.126 (0.098 - 0.159) |
| L2 | 10 | 10 | 3 | 0.432 (0.388 - 0.476) | 0.196 (0.162 - 0.234) |
| L2 | 20 | 10 | 3 | 0.582 (0.537 - 0.625) | 0.288 (0.249 - 0.330) |
| L2 | 30 | 10 | 3 | 0.684 (0.640 - 0.724) | 0.352 (0.310 - 0.395) |
| L2 | 40 | 10 | 3 | 0.702 (0.659 - 0.741) | 0.356 (0.314 - 0.399) |
| L2 | 10 | 0 | 3 | 0.010 (0.003 - 0.024) | 0.000 (0.000 - 0.009) |
| L2 | 10 | 20 | 3 | 0.812 (0.774 - 0.844) | 0.570 (0.525 - 0.613) |
| L2 | 10 | 30 | 3 | 0.832 (0.795 - 0.863) | 0.688 (0.645 - 0.728) |
| L2 | 10 | 10 | 1 | 1.000 (0.990 - 1.000) | 0.876 (0.843 - 0.902) |
| L2 | 10 | 10 | 2 | 0.688 (0.645 - 0.728) | 0.512 (0.467 - 0.556) |
| L2 | 10 | 10 | 3 | 0.464 (0.419 - 0.508) | 0.224 (0.188 - 0.263) |
| L2 | 10 | 10 | 4 | 0.272 (0.233 - 0.313) | 0.138 (0.109 - 0.172) |
| L2 | 10 | 10 | 5 | 0.284 (0.245 - 0.326) | 0.192 (0.158 - 0.229) |
| L3 | 10 | 10 | 3 | 0.488 (0.443 - 0.532) | 0.226 (0.190 - 0.265) |
| L3 | 20 | 10 | 3 | 0.606 (0.561 - 0.648) | 0.312 (0.271 - 0.354) |
| L3 | 30 | 10 | 3 | 0.666 (0.622 - 0.706) | 0.330 (0.289 - 0.373) |
| L3 | 40 | 10 | 3 | 0.862 (0.827 - 0.890) | 0.508 (0.463 - 0.552) |
| L3 | 10 | 0 | 3 | 0.008 (0.002 - 0.021) | 0.004 (0.000 - 0.015) |
| L3 | 10 | 20 | 3 | 0.816 (0.778 - 0.848) | 0.572 (0.527 - 0.615) |
| L3 | 10 | 30 | 3 | 0.820 (0.782 - 0.852) | 0.670 (0.626 - 0.710) |
| L3 | 10 | 10 | 1 | 1.000 (0.990 - 1.000) | 0.848 (0.812 - 0.877) |
| L3 | 10 | 10 | 2 | 0.706 (0.663 - 0.745) | 0.556 (0.511 - 0.599) |
| L3 | 10 | 10 | 3 | 0.472 (0.427 - 0.516) | 0.242 (0.205 - 0.282) |
| L3 | 10 | 10 | 4 | 0.310 (0.270 - 0.352) | 0.126 (0.098 - 0.159) |
| L3 | 10 | 10 | 5 | 0.234 (0.198 - 0.274) | 0.160 (0.129 - 0.195) |
| L4 | 10 | 10 | 3 | 0.456 (0.411 - 0.500) | 0.204 (0.170 - 0.242) |
| L4 | 20 | 10 | 3 | 0.602 (0.557 - 0.644) | 0.332 (0.291 - 0.375) |
| L4 | 30 | 10 | 3 | 0.614 (0.569 - 0.650) | 0.306 (0.266 - 0.348) |
| L4 | 40 | 10 | 3 | 0.696 (0.653 - 0.735) | 0.326 (0.285 - 0.369) |
| L4 | 10 | 0 | 3 | 0.004 (0.000 - 0.015) | 0.000 (0.000 - 0.009) |
| L4 | 10 | 20 | 3 | 0.818 (0.780 - 0.850) | 0.580 (0.535 - 0.623) |
| L4 | 10 | 30 | 3 | 0.825 (0.788 - 0.857) | 0.677 (0.634 - 0.717) |
| L4 | 10 | 10 | 1 | 1.000 (0.990 - 1.000) | 0.840 (0.804 - 0.870) |
| L4 | 10 | 10 | 2 | 0.664 (0.620 - 0.704) | 0.502 (0.457 - 0.546) |
| L4 | 10 | 10 | 3 | 0.428 (0.384 - 0.472) | 0.192 (0.158 - 0.229) |
| L4 | 10 | 10 | 4 | 0.288 (0.249 - 0.330) | 0.140 (0.111 - 0.174) |
| L4 | 10 | 10 | 5 | 0.216 (0.181 - 0.255) | 0.150 (0.120 - 0.185) |
| L5 | 10 | 10 | 3 | 0.466 (0.421 - 0.510) | 0.210 (0.175 - 0.248) |
| L5 | 20 | 10 | 3 | 0.603 (0.585 - 0.672) | 0.310 (0.270 - 0.352) |
| L5 | 30 | 10 | 3 | 0.608 (0.563 - 0.650) | 0.350 (0.308 - 0.393) |
| L5 | 40 | 10 | 3 | 0.690 (0.647 - 0.729) | 0.326 (0.285 - 0.369) |
| L5 | 10 | 0 | 3 | 0.004 (0.000 - 0.015) | 0.002 (0.000 - 0.012) |
| L5 | 10 | 20 | 3 | 0.800 (0.761 - 0.833) | 0.572 (0.527 - 0.615) |
| L5 | 10 | 30 | 3 | 0.858 (0.823 - 0.886) | 0.734 (0.692 - 0.771) |
| L5 | 10 | 10 | 1 | 1.000 (0.990 - 1.000) | 0.846 (0.810 - 0.875) |
| L5 | 10 | 10 | 2 | 0.710 (0.667 - 0.749) | 0.532 (0.487 - 0.576) |
| L5 | 10 | 10 | 3 | 0.484 (0.439 - 0.528) | 0.254 (0.216 - 0.294) |
| L5 | 10 | 10 | 4 | 0.316 (0.275 - 0.359) | 0.128 (0.100 - 0.161) |
| L5 | 10 | 10 | 5 | 0.206 (0.171 - 0.244) | 0.146 (0.116 - 0.180) |
| L6 | 10 | 10 | 3 | 0.452 (0.407 - 0.496) | 0.208 (0.173 - 0.246) |
| L6 | 20 | 10 | 3 | 0.610 (0.565 - 0.652) | 0.296 (0.256 - 0.338) |
| L6 | 30 | 10 | 3 | 0.628 (0.583 - 0.670) | 0.330 (0.289 - 0.373) |
| L6 | 40 | 10 | 3 | 0.636 (0.591 - 0.677) | 0.304 (0.264 - 0.346) |
| L6 | 10 | 0 | 3 | 0.002 (0.000 - 0.012) | 0.000 (0.000 - 0.009) |
| L6 | 10 | 20 | 3 | 0.836 (0.799 - 0.866) | 0.624 (0.579 - 0.666) |
| L6 | 10 | 30 | 3 | 0.846 (0.810 - 0.875) | 0.736 (0.694 - 0.773) |
| L6 | 10 | 10 | 1 | 1.000 (0.990 - 1.000) | 0.860 (0.825 - 0.888) |
| L6 | 10 | 10 | 2 | 0.658 (0.614 - 0.699) | 0.510 (0.465 - 0.554) |
| L6 | 10 | 10 | 3 | 0.422 (0.378 - 0.466) | 0.216 (0.181 - 0.255) |
| L6 | 10 | 10 | 4 | 0.310 (0.270 - 0.352) | 0.154 (0.124 - 0.189) |
| L6 | 10 | 10 | 5 | 0.234 (0.198 - 0.274) | 0.170 (0.138 - 0.206) |
| L7 | 10 | 10 | 3 | 0.494 (0.449 - 0.538) | 0.258 (0.220 - 0.299) |
| L7 | 20 | 10 | 3 | 0.610 (0.565 - 0.652) | 0.288 (0.249 - 0.330) |
| L7 | 30 | 10 | 3 | 0.652 (0.608 - 0.693) | 0.310 (0.270 - 0.352) |
| L7 | 40 | 10 | 3 | 0.666 (0.622 - 0.706) | 0.346 (0.304 - 0.389) |
| L7 | 10 | 0 | 3 | 0.008 (0.002 - 0.021) | 0.000 (0.000 - 0.009) |
| L7 | 10 | 20 | 3 | 0.786 (0.746 - 0.820) | 0.560 (0.515 - 0.603) |
| L7 | 10 | 30 | 3 | 0.822 (0.784 - 0.853) | 0.700 (0.657 - 0.739) |
| L7 | 10 | 10 | 1 | 1.000 (0.990 - 1.000) | 0.886 (0.854 - 0.911) |
| L7 | 10 | 10 | 2 | 0.666 (0.622 - 0.706) | 0.484 (0.439 - 0.528) |
| L7 | 10 | 10 | 3 | 0.466 (0.421 - 0.510) | 0.242 (0.205 - 0.282) |
| L7 | 10 | 10 | 4 | 0.288 (0.249 - 0.330) | 0.108 (0.082 - 0.139) |
| L7 | 10 | 10 | 5 | 0.274 (0.235 - 0.315) | 0.186 (0.153 - 0.223) |
| L8 | 10 | 10 | 3 | 0.484 (0.439 - 0.528) | 0.264 (0.226 - 0.305) |
| L8 | 20 | 10 | 3 | 0.590 (0.545 - 0.633) | 0.310 (0.270 - 0.352) |
| L8 | 30 | 10 | 3 | 0.640 (0.595 - 0.681) | 0.322 (0.281 - 0.365) |
| L8 | 40 | 10 | 3 | 0.670 (0.626 - 0.710) | 0.382 (0.339 - 0.426) |
| L8 | 10 | 0 | 3 | 0.020 (0.010 - 0.037) | 0.002 (0.000 - 0.012) |
| L8 | 10 | 20 | 3 | 0.836 (0.799 - 0.866) | 0.598 (0.553 - 0.641) |
| L8 | 10 | 30 | 3 | 0.890 (0.858 - 0.915) | 0.732 (0.690 - 0.769) |
| L8 | 10 | 10 | 1 | 1.000 (0.990 - 1.000) | 0.870 (0.836 - 0.897) |
| L8 | 10 | 10 | 2 | 0.658 (0.614 - 0.699) | 0.504 (0.459 - 0.548) |
| L8 | 10 | 10 | 3 | 0.432 (0.388 - 0.476) | 0.242 (0.205 - 0.282) |
| L8 | 10 | 10 | 4 | 0.304 (0.264 - 0.346) | 0.156 (0.125 - 0.191) |
| L8 | 10 | 10 | 5 | 0.250 (0.213 - 0.290) | 0.168 (0.136 - 0.204) |
| L9 | 10 | 10 | 3 | 0.456 (0.411 - 0.500) | 0.206 (0.171 - 0.244) |
| L9 | 20 | 10 | 3 | 0.580 (0.535 - 0.623) | 0.288 (0.249 - 0.330) |
| L9 | 30 | 10 | 3 | 0.666 (0.622 - 0.706) | 0.326 (0.285 - 0.369) |
| L9 | 40 | 10 | 3 | 0.712 (0.669 - 0.750) | 0.338 (0.296 - 0.387) |
| L9 | 10 | 0 | 3 | 0.006 (0.001 - 0.018) | 0.002 (0.000 - 0.012) |
| L9 | 10 | 20 | 3 | 0.820 (0.782 - 0.852) | 0.614 (0.569 - 0.650) |
| L9 | 10 | 30 | 3 | 0.832 (0.795 - 0.863) | 0.706 (0.663 - 0.745) |
| L9 | 10 | 10 | 1 | 1.000 (0.990 - 1.000) | 0.852 (0.881 - 0.049) |
| L9 | 10 | 10 | 2 | 0.700 (0.657 - 0.739) | 0.512 (0.467 - 0.556) |
| L9 | 10 | 10 | 3 | 0.438 (0.394 - 0.482) | 0.224 (0.188 - 0.263) |
| L9 | 10 | 10 | 4 | 0.308 (0.268 - 0.350) | 0.148 (0.118 - 0.182) |
| L9 | 10 | 10 | 5 | 0.208 (0.173 - 0.246) | 0.142 (0.113 - 0.176) |
| L10 | 10 | 10 | 3 | 0.466 (0.421 - 0.510) | 0.208 (0.173 - 0.246) |
| L10 | 20 | 10 | 3 | 0.582 (0.537 - 0.625) | 0.274 (0.235 - 0.315) |
| L10 | 30 | 10 | 3 | 0.672 (0.628 - 0.712) | 0.326 (0.285 - 0.369) |
| L10 | 40 | 10 | 3 | 0.692 (0.649 - 0.731) | 0.316 (0.275 - 0.359) |
| L10 | 10 | 0 | 3 | 0.010 (0.003 - 0.024) | 0.002 (0.000 - 0.012) |
| L10 | 10 | 20 | 3 | 0.826 (0.789 - 0.857) | 0.534 (0.489 - 0.578) |
| L10 | 10 | 30 | 3 | 0.864 (0.830 - 0.892) | 0.651 (0.606 - 0.691) |
| L10 | 10 | 10 | 1 | 1.000 (0.990 - 1.000) | 0.840 (0.870 - 0.047) |
| L10 | 10 | 10 | 2 | 0.650 (0.606 - 0.691) | 0.486 (0.441 - 0.530) |
| L10 | 10 | 10 | 3 | 0.492 (0.447 - 0.536) | 0.242 (0.205 - 0.282) |
| L10 | 10 | 10 | 4 | 0.304 (0.264 - 0.346) | 0.158 (0.127 - 0.193) |
| L10 | 10 | 10 | 5 | 0.228 (0.192 - 0.267) | 0.144 (0.115 - 0.178) |

R codes together with life history information used for the simulations in R package AlleleRetain. We provided an example for subpopulation L10. R codes and definition of each term were obtained from [49].

aR.temp = aRetain (q0 = 0.05, sourceN = 440, startN = 20, startAge = "adult",

startSR = 0.5, exactSR= FALSE, inisurv = 0.90, addN = 0, addyrs = c (0),

migrN = 0, migrfreq = 0, mpriority = FALSE, K = 440,

Klag = 0, KAdults = FALSE, reprolag = 0, mature = 2, matingSys =

"polygyny", matingLength = "seasonal", meanMLRS = 1, sdMLRS = 0,

reproAgeM = c (2:5), AgeOnMLRS = "age/age", nMatings = 1,

MaxAge = 5, SenesAge = 4, adsurvivalF = 0.85,

adsurvivalM = 0.95, nonbrsurv = 0.70, nonbrsurvK = 0.70, juvsurv =

0.60, juvsurvK = 0.60, youngperF = 1.64, SDypF = 0.25, ypF1 = 1, ypF1yr

= 1, MAXypF = 1.64, MAXypFK = 1.64, ypFsex = "female", youngSR = 0.5,

GeneCount = "adult", nyears = 10, nrepl = 1000,

nreplprint = 10, printplots = FALSE)

aRetain.summary (aR.temp,GeneCount = "all", alpha=0.05, dropextinct = TRUE)

where,

q0 = proportion of rare alleles,

sourceN = population size,

startN = initial number of translocated individuals,

startAge = age group,

startSR = sex ratio,

exactSR = denotes if sex ratio is true or obtained randomly based on startSR,

inisurv = initial survival rate,

addN = number of individuals to be added to established population,

addyrs = interval (year 1, 2 and so on) at which addN will occur,

migN = number of assisted migrants after translocation,

migrfreq = frequency at which assisted migrants were translocated,

mpriority = migrants recruitment (true or false),

K = carrying capacity of new population,

KAdults = true (adult) or false (adult, sub-adult or juveniles),

reprolag = number of years when reproduction = 0,

mature = age at sexual maturity,

matingSys = sexual behavior,

matingLength = fidelity or divorce per annum,

meanMLRS = mean lifetime reproductive success (LRS) of males,

sdMLRS = standard deviation of LRS for males,

reproAgeM = age of sexual maturity of males,

AgeOnMLRS = LRS for males in relation to reproAgeM,

nMatings = mean number of matings of females per annum,

MaxAge = maximum life expectency,

SenesAge = senescence age,

adsurvivalF = survival rate of adult females per annum,

adsurvivalM = survival rate of adult males per annum,

nonbrsurv = survival rate of non-breeding individuals per annum,

nonbrsurvK = survival rate of non breeding individuals per annum when population size = K,

juvsurv = first year survival rate of juvenile when population < K,

juvsurvK = first year survival rate per annum of juvenile when population = K,

youngperF = average number of young observed per annum,

SDypF = standard deviation of youngperF,

ypF1 = reproductive success of early breeders,

ypF1yr = age at which ypF1 occurs,

MAXypF = maximum number of young produced on a year basic per individual,

MAXypFK = maximum number of young produced on a year basic per individual when population = K,

ypFsex = annual reproductive output affected by male, female or both,

youngSR = frequency of male offspring,

GeneCount = simulations encompassing either all age groups or only breeding adults

nyears = number of years at which simulations will be conducted (e.g. 50 years in this study)

nrepl = number of iterations,

nreplprint = number of iterations at which results are illustrated, and

printplots = plotting specific parameters.
