# Supplementary material for: High Risks of Losing Genetic Diversity in an Endemic Mauritian Gecko: Implications for Conservation
Source: PLoS One. 2014 Jun 25;9(6):e93387. doi: 10.1371/journal.pone.0093387 (PMC4070904; doi:10.1371/journal.pone.0093387)
Supplement: Table S2 — Mitochondrial DNA primers in Phelsuma guimbeaui. (DOC) [file pone.0093387.s002.doc]

**Table S2. Mitochondrial DNA primers in *Phelsuma guimbeaui*.**

| **Primer** | **Length (bp)** | **Primer melting temperature (^o^C)*** | **PCR annealing temperature (^o^C)** | **Sequence (5'-3")** |
| --- | --- | --- | --- | --- |
| Pgu-*cytb*_F | 20 | 59.7 | 59.0 | GGCTCATTACTCGGCCTATG |
| Pgu-*cytb*_R | 23 | 59.7 | 59.0 | GATGGAGAAGTATGGGTGAAATG |
| Pgu-*6SrRNA*_F | 26 | 55.2 | 59.0 | CTTCTAAATAGAGACTTGTATGAACG |
| Pgu-*6SrRNA*_R | 19 | 55.4 | 59.0 | CACTTGGAAGTCCTGATCC |

*calculated using Primer3 software
