# Supplementary material for: High Risks of Losing Genetic Diversity in an Endemic Mauritian Gecko: Implications for Conservation
Source: PLoS One. 2014 Jun 25;9(6):e93387. doi: 10.1371/journal.pone.0093387 (PMC4070904; doi:10.1371/journal.pone.0093387)
Supplement: Table S3 — Screening for data quality to select the best loci for analyses of molecular variation in Phelsuma guimbeaui. (DOC) [file pone.0093387.s003.doc]

***Table S3. Screening for data quality to select the best loci for analyses of molecular variation in Phelsuma guimbeaui. Table continues next page.***

| **Locus** | **EMBL accession**  **number** | **Amplification success rate**  **(%)** | **Error rate per allele** | **One base jump** | **Loci under selection** | **Alleles larger than 500bp** |
| --- | --- | --- | --- | --- | --- | --- |
| Pgu001 | HF567457 | 99.6 | 0.000 | Yes | Yes | No |
| Pgu004 | HF567460 | 98.5 | 0.000 | No | Yes | No |
| Pgu005 | HF567461 | 94.2 | 0.000 | No | No | No |
| Pgu006 | HF567462 | 98.5 | 0.000 | Yes | No | Yes |
| Pgu007 | HF567463 | 94.6 | 0.000 | No | No | No |
| Pgu008 | HF567464 | 99.2 | 0.000 | No | Yes | Yes |
| Pgu009 | HF567465 | 96.9 | 0.018 | No | No | No |
| Pgu010 | HF567466 | 99.2 | 0.000 | No | No | No |
| Pgu011 | HF567467 | 98.1 | 0.000 | Yes | No | No |
| Pgu012 | HF567468 | 97.3 | 0.000 | No | No | No |
| Pgu014 | HF567470 | 94.2 | 0.000 | No | No | No |
| Pgu015 | HF567471 | 100.0 | 0.000 | No | Yes | Yes |
| Pgu016 | HF567472 | 96.9 | 0.000 | No | No | No |
| Pgu017 | HF567473 | 99.2 | 0.016 | No | No | No |
| Pgu018 | HF567474 | 95.8 | 0.000 | No | No | Yes |
| Pgu019 | HF567475 | 97.3 | 0.000 | No | No | No |
| Pgu020 | HF567476 | 99.6 | 0.000 | No | Yes | No |
| Pgu021 | HF567477 | 98.1 | 0.000 | No | No | No |
| Pgu022 | HF567478 | 98.8 | 0.000 | No | No | No |
| Pgu023 | HF567479 | 96.9 | 0.000 | No | No | Yes |
| Pgu024 | HF567480 | 97.3 | 0.000 | No | Yes | No |
| Pgu025 | HF567481 | 99.2 | 0.000 | No | No | No |
| Pgu026 | HF567482 | 98.5 | 0.000 | Yes | No | No |
| Pgu027 | HF567483 | 83.0 | 0.000 | No | No | No |
| Pgu028 | HF567484 | 86.5 | 0.000 | No | Yes | No |
| Pgu029 | HF567485 | 87.3 | 0.000 | No | No | No |
| Pgu030 | HF567486 | 96.1 | 0.000 | Yes | Yes | No |
| Pgu031 | HF567487 | 98.8 | 0.000 | No | Yes | No |
| Pgu032 | HF567488 | 95.8 | 0.000 | No | No | No |
| Pgu033 | HF567489 | 96.5 | 0.000 | No | No | No |
| Pgu034 | HF567490 | 87.3 | 0.000 | No | No | No |
| Pgu035 | HF567491 | 92.3 | 0.000 | No | Yes | No |
| Pgu036 | HF567492 | 96.5 | 0.000 | No | No | No |
| Pgu037 | HF567493 | 97.3 | 0.000 | No | Yes | No |
| Pgu038 | HF567494 | 98.1 | 0.000 | Yes | No | No |
| Pgu039 | HF567495 | 96.1 | 0.000 | No | No | No |
| Pgu040 | HF567496 | 97.7 | 0.033 | No | Yes | No |
| Pgu041 | HF567497 | 96.9 | 0.000 | No | No | No |
| Pgu042 | HF567498 | 96.1 | 0.000 | No | No | No |
| Pgu043 | HF567499 | 57.6 | 0.000 | No | No | Yes |
| Pgu044 | HF567500 | 95.4 | 0.000 | No | No | No |
