# Supplementary material for: High Risks of Losing Genetic Diversity in an Endemic Mauritian Gecko: Implications for Conservation
Source: PLoS One. 2014 Jun 25;9(6):e93387. doi: 10.1371/journal.pone.0093387 (PMC4070904; doi:10.1371/journal.pone.0093387)
Supplement: Table S4 — The number of subpopulations of Phelsuma guimbeaui not in Hardy-Weinberg equilibrium (P<0.05) at the different loci. (DOC) [file pone.0093387.s004.doc]

**Table S4.** The number of subpopulations of *Phelsuma guimbeaui* not in Hardy-Weinberg equilibrium (P <0.05) at the different loci.

*Table continues next page.*

| **Locus** | **EMBL**  **accession**  **number** | **Subpopulations**  **L1 L2 L3 L4 L5 L6 L7 L8 L9 L10** | | | | | | | | | | **Number of**  **subpopulations**  **(P <0.05)** |
| --- | --- | --- | --- | --- | --- | --- | --- | --- | --- | --- | --- | --- |
| Pgu001 | HF567457 | 0.001 | 0.012 | 0.023 | 0.352 | 0.224 | 0.002 | 0.013 | 0.102 | 0.213 | 0.001 | 6 |
| Pgu004 | HF567460 | 0.633 | 0.062 | 0.007 | 0.006 | 0.045 | 0.763 | 0.004 | 0.851 | 0.045 | 0.034 | 6 |
| Pgu006 | HF567462 | 0.091 | 0.007 | 0.173 | 0.804 | 0.002 | 0.297 | 0.256 | 0.605 | 0.724 | 0.677 | 2 |
| Pgu007 | HF567463 | 0.756 | 0.432 | 0.017 | 0.007 | 0.185 | 0.011 | 0.047 | 0.157 | 0.623 | 0.684 | 4 |
| Pgu008 | HF567464 | 0.003 | 0.629 | 0.007 | 0.046 | 0.207 | 0.517 | 0.118 | 0.013 | 0.014 | 0.483 | 5 |
| Pgu009 | HF567465 | 1.000 | 0.002 | 0.948 | 0.707 | 0.832 | 0.556 | 0.179 | 0.568 | 0.932 | 0.087 | 1 |
| Pgu010 | HF567466 | 0.282 | 0.670 | 0.997 | 0.939 | 0.604 | 0.567 | 0.707 | 0.927 | 0.492 | 0.834 | 0 |
| Pgu011 | HF567467 | 0.454 | 0.001 | 0.523 | 0.167 | 0.812 | 0.183 | 0.786 | 0.115 | 0.994 | 0.237 | 1 |
| Pgu012 | HF567468 | 0.602 | 0.571 | 0.664 | 0.535 | 0.523 | 0.517 | 0.927 | 0.935 | 0.272 | 0.099 | 0 |
| Pgu014 | HF567470 | 0.922 | 0.516 | 0.077 | 0.024 | 0.002 | 0.982 | 0.402 | 0.157 | 0.657 | 0.014 | 3 |
| Pgu015 | HF567471 | 0.719 | 0.068 | 0.212 | 0.075 | 0.457 | 0.007 | 0.193 | 0.058 | 0.342 | 0.694 | 1 |
| Pgu016 | HF567472 | 0.001 | 0.133 | 0.754 | 0.162 | 0.402 | 0.009 | 0.227 | 0.105 | 0.607 | 0.254 | 2 |
| Pgu017 | HF567473 | 0.016 | 0.244 | 0.943 | 0.854 | 0.987 | 0.435 | 0.122 | 0.199 | 0.605 | 0.333 | 1 |
| Pgu018 | HF567474 | 0.052 | 0.075 | 0.024 | 0.023 | 0.003 | 0.023 | 0.011 | 0.004 | 0.007 | 0.017 | 8 |
| Pgu019 | HF567475 | 0.238 | 0.221 | 0.402 | 0.137 | 0.007 | 0.774 | 0.273 | 0.235 | 0.526 | 0.337 | 1 |
| Pgu020 | HF567476 | 0.350 | 0.335 | 0.557 | 0.717 | 0.092 | 0.577 | 0.865 | 0.603 | 0.691 | 0.133 | 0 |
| Pgu021 | HF567477 | 0.576 | 0.657 | 0.254 | 0.467 | 0.207 | 0.297 | 0.165 | 0.967 | 0.358 | 0.374 | 0 |
| Pgu022 | HF567478 | 0.407 | 0.502 | 0.174 | 0.353 | 0.552 | 0.869 | 0.252 | 0.033 | 0.174 | 0.004 | 2 |
| Pgu023 | HF567479 | 0.005 | 0.044 | 0.007 | 0.055 | 0.004 | 0.017 | 0.002 | 0.353 | 0.022 | 0.025 | 8 |
| Pgu024 | HF567480 | 0.096 | 0.593 | 0.378 | 0.977 | 0.712 | 0.503 | 0.733 | 0.114 | 0.785 | 0.015 | 1 |
| Pgu025 | HF567481 | 0.031 | 0.224 | 0.007 | 0.464 | 0.164 | 0.791 | 0.043 | 0.314 | 0.653 | 0.878 | 3 |
| Pgu026 | HF567482 | 0.056 | 0.002 | 0.004 | 0.227 | 0.252 | 0.001 | 0.018 | 0.985 | 0.001 | 0.377 | 5 |
| Pgu027 | HF567483 | 0.274 | 0.504 | 0.007 | 0.590 | 0.014 | 0.167 | 0.095 | 0.142 | 0.661 | 0.847 | 2 |
| Pgu028 | HF567484 | 0.076 | 0.047 | 0.819 | 0.110 | 0.272 | 0.154 | 0.410 | 0.237 | 0.042 | 0.464 | 2 |
| Pgu029 | HF567485 | 0.955 | 0.255 | 0.637 | 0.412 | 0.505 | 0.828 | 0.850 | 0.926 | 0.057 | 0.610 | 0 |
| Pgu030 | HF567486 | 0.136 | 0.497 | 0.945 | 0.844 | 0.023 | 0.817 | 0.004 | 0.061 | 0.045 | 0.054 | 3 |
| Pgu031 | HF567487 | 0.534 | 0.739 | 1.004 | 1.000 | 0.241 | 0.925 | 1.000 | 0.771 | 0.058 | 0.138 | 1 |
| Pgu032 | HF567488 | 0.446 | 0.327 | 0.927 | 0.455 | 0.016 | 0.134 | 0.828 | 0.171 | 0.995 | 0.278 | 1 |
| Pgu034 | HF567490 | 0.207 | 0.135 | 0.009 | 0.002 | 0.046 | 0.663 | 0.208 | 0.708 | 0.595 | 0.072 | 3 |
| Pgu035 | HF567491 | 0.007 | 0.007 | 0.007 | 0.002 | 0.007 | 0.001 | 0.003 | 0.004 | 0.003 | 0.024 | 10 |
| Pgu036 | HF567492 | 0.453 | 0.353 | 0.541 | 0.102 | 0.195 | 0.348 | 0.521 | 0.655 | 0.354 | 0.083 | 0 |
| Pgu037 | HF567493 | 0.481 | 0.297 | 0.007 | 0.048 | 0.557 | 0.302 | 0.590 | 0.209 | 0.794 | 0.014 | 3 |
| Pgu038 | HF567494 | 0.217 | 0.705 | 0.535 | 0.828 | 0.003 | 0.438 | 0.260 | 0.183 | 0.557 | 0.494 | 1 |
| Pgu039 | HF567495 | 0.067 | 0.387 | 0.178 | 0.012 | 0.017 | 0.557 | 0.009 | 0.008 | 0.002 | 0.007 | 6 |
| Pgu040 | HF567496 | 0.624 | 0.762 | 0.027 | 0.884 | 0.826 | 0.844 | 0.464 | 0.658 | 0.409 | 0.067 | 1 |
| Pgu041 | HF567497 | 0.021 | 0.307 | 0.003 | 0.681 | 0.862 | 0.973 | 0.026 | 0.352 | 0.755 | 0.405 | 3 |
| Pgu042 | HF567498 | 0.152 | 0.442 | 0.047 | 0.894 | 0.977 | 0.202 | 0.094 | 0.202 | 0.092 | 0.000 | 2 |
| Pgu044 | HF567500 | 0.867 | 0.793 | 0.724 | 0.333 | 0.081 | 0.321 | 0.153 | 0.282 | 0.325 | 0.287 | 0 |
