# Supplementary material for: High Risks of Losing Genetic Diversity in an Endemic Mauritian Gecko: Implications for Conservation
Source: PLoS One. 2014 Jun 25;9(6):e93387. doi: 10.1371/journal.pone.0093387 (PMC4070904; doi:10.1371/journal.pone.0093387)
Supplement: Table S5 — Null allele frequencies in ten subpopulations of Phelsuma guimbeaui. (DOC) [file pone.0093387.s005.doc]

**Table S5.** Null allele frequencies in ten subpopulations of *Phelsuma guimbeaui*. *Table continues next page.*

| **Locus** | **EMBL accession**  **number** | **Subpopulations**  **L1 L2 L3 L4 L5 L6 L7 L8 L9 L10** | | | | | | | | | | **TNSP*** |
| --- | --- | --- | --- | --- | --- | --- | --- | --- | --- | --- | --- | --- |
| Pgu001 | HF567457 | 0.281 | 0.062 | 0.094 | 0.046 | 0.055 | 0.195 | 0.133 | 0.112 | 0.006 | 0.382 | 5 |
| Pgu004 | HF567460 | -0.055 | -0.023 | 0.033 | 0.100 | 0.032 | -0.014 | 0.072 | -0.019 | 0.044 | 0.063 | 0 |
| Pgu006 | HF567462 | 0.025 | 0.125 | 0.052 | 0.004 | 0.071 | 0.019 | 0.018 | 0.017 | -0.013 | -0.038 | 1 |
| Pgu007 | HF567463 | -0.053 | 0.028 | 0.081 | 0.141 | 0.037 | 0.157 | 0.096 | 0.026 | -0.035 | -0.019 | 2 |
| Pgu008 | HF567464 | 0.176 | 0.022 | 0.133 | 0.089 | 0.036 | -0.013 | 0.037 | 0.034 | 0.064 | 0.032 | 2 |
| Pgu009 | HF567465 | -0.052 | 0.136 | -0.028 | -0.048 | -0.023 | 0.004 | 0.093 | -0.022 | -0.026 | 0.047 | 1 |
| Pgu010 | HF567466 | 0.046 | 0.009 | -0.034 | -0.043 | 0.014 | -0.012 | -0.072 | -0.036 | -0.048 | -0.042 | 0 |
| Pgu011 | HF567467 | 0.008 | 0.082 | -0.046 | 0.043 | -0.053 | 0.023 | -0.043 | -0.035 | -0.028 | -0.013 | 0 |
| Pgu012 | HF567468 | -0.029 | -0.023 | -0.054 | 0.023 | 0.016 | -0.012 | -0.074 | -0.044 | 0.043 | 0.015 | 0 |
| Pgu014 | HF567470 | -0.072 | 0.006 | -0.012 | 0.073 | 0.270 | -0.046 | 0.003 | 0.058 | -0.023 | 0.062 | 1 |
| Pgu015 | HF567471 | -0.013 | 0.071 | 0.014 | 0.021 | 0.061 | 0.054 | -0.014 | 0.032 | 0.031 | -0.039 | 0 |
| Pgu016 | HF567472 | 0.171 | 0.100 | -0.076 | 0.067 | -0.022 | 0.193 | 0.056 | -0.032 | 0.035 | -0.094 | 2 |
| Pgu017 | HF567473 | 0.105 | -0.017 | -0.011 | -0.039 | -0.035 | -0.053 | 0.048 | 0.012 | -0.052 | -0.023 | 1 |
| Pgu018 | HF567474 | 0.054 | 0.013 | 0.062 | 0.092 | 0.246 | 0.077 | 0.089 | 0.184 | 0.093 | 0.077 | 2 |
| Pgu019 | HF567475 | -0.066 | 0.011 | 0.058 | 0.044 | 0.100 | 0.012 | -0.051 | 0.082 | 0.034 | 0.063 | 0 |
| Pgu020 | HF567476 | 0.027 | 0.058 | -0.032 | -0.073 | 0.100 | -0.012 | -0.043 | -0.034 | -0.129 | -0.065 | 0 |
| Pgu021 | HF567477 | -0.028 | 0.012 | 0.037 | 0.007 | 0.089 | 0.033 | -0.064 | 0.013 | 0.015 | 0.132 | 1 |
| Pgu022 | HF567478 | 0.006 | 0.048 | 0.036 | 0.122 | 0.013 | 0.054 | -0.056 | 0.152 | 0.045 | 0.246 | 3 |
| Pgu023 | HF567479 | 0.267 | 0.077 | 0.184 | 0.100 | 0.324 | 0.112 | 0.148 | 0.014 | 0.154 | 0.044 | 6 |
| Pgu024 | HF567480 | 0.033 | -0.053 | 0.107 | -0.026 | -0.012 | -0.054 | -0.036 | 0.113 | 0.062 | 0.182 | 3 |
| Pgu025 | HF567481 | 0.114 | 0.015 | 0.196 | 0.017 | 0.026 | -0.023 | 0.099 | -0.073 | -0.033 | 0.064 | 2 |
| Pgu026 | HF567482 | 0.022 | 0.367 | 0.124 | 0.156 | 0.064 | 0.345 | 0.067 | 0.011 | 0.171 | 0.053 | 5 |
| Pgu027 | HF567483 | 0.082 | 0.039 | 0.246 | -0.048 | 0.102 | 0.029 | 0.086 | 0.112 | 0.002 | 0.004 | 3 |
| Pgu028 | HF567484 | 0.018 | 0.184 | 0.014 | 0.089 | 0.083 | 0.024 | -0.124 | 0.134 | 0.059 | 0.062 | 2 |
| Pgu029 | HF567485 | -0.041 | 0.005 | 0.006 | -0.084 | 0.084 | -0.072 | 0.003 | -0.050 | -0.054 | -0.034 | 0 |
| Pgu030 | HF567486 | 0.033 | 0.026 | -0.034 | -0.016 | 0.056 | -0.013 | 0.111 | 0.030 | -0.012 | -0.015 | 1 |
| Pgu031 | HF567487 | -0.025 | -0.107 | -0.052 | -0.044 | 0.054 | 0.021 | -0.032 | 0.022 | 0.166 | 0.154 | 2 |
| Pgu032 | HF567488 | -0.118 | 0.002 | -0.047 | 0.053 | 0.153 | 0.005 | 0.054 | 0.053 | -0.012 | 0.009 | 1 |
| Pgu034 | HF567490 | 0.051 | 0.099 | 0.073 | 0.207 | 0.068 | 0.001 | 0.059 | -0.036 | -0.024 | 0.074 | 1 |
| Pgu035 | HF567491 | 0.402 | 0.381 | 0.255 | 0.469 | 0.349 | 0.349 | 0.172 | 0.304 | 0.323 | 0.236 | 10 |
| Pgu036 | HF567492 | -0.014 | -0.016 | -0.027 | -0.012 | 0.024 | -0.010 | 0.024 | 0.018 | 0.011 | 0.082 | 0 |
| Pgu037 | HF567493 | 0.092 | 0.011 | 0.266 | 0.170 | 0.053 | 0.014 | 0.039 | 0.044 | 0.015 | 0.143 | 3 |
| Pgu038 | HF567494 | 0.077 | -0.058 | 0.014 | -0.050 | 0.044 | -0.049 | 0.021 | 0.100 | -0.062 | -0.021 | 0 |
| Pgu039 | HF567495 | 0.100 | -0.029 | 0.092 | 0.080 | 0.042 | 0.017 | 0.349 | 0.558 | 0.389 | 0.346 | 4 |
| Pgu040 | HF567496 | -0.119 | 0.011 | 0.174 | -0.053 | -0.022 | -0.056 | -0.051 | -0.089 | -0.044 | 0.084 | 1 |
| Pgu041 | HF567497 | 0.122 | -0.016 | 0.259 | 0.045 | 0.026 | -0.011 | -0.033 | 0.034 | -0.036 | -0.063 | 2 |
| Pgu042 | HF567498 | -0.059 | 0.011 | 0.047 | -0.043 | -0.034 | 0.060 | 0.067 | -0.039 | 0.012 | 0.018 | 0 |
| Pgu044 | HF567500 | -0.029 | 0.012 | -0.038 | 0.030 | 0.118 | 0.026 | 0.095 | 0.017 | 0.019 | -0.039 | 1 |

*TNSP: total number of subpopulations showing evidence of null alleles (frequency >0.1).
