# Supplementary material for: High Risks of Losing Genetic Diversity in an Endemic Mauritian Gecko: Implications for Conservation
Source: PLoS One. 2014 Jun 25;9(6):e93387. doi: 10.1371/journal.pone.0093387 (PMC4070904; doi:10.1371/journal.pone.0093387)
Supplement: Table S6 — Number of alleles observed for the 20 loci used in the microsatellite analyses of Phelsuma guimbeaui. (DOC) [file pone.0093387.s006.doc]

**Table S6. Number of alleles observed for the 20 loci used in the microsatellite analyses of *Phelsuma guimbeaui*.**

| **Locus** | **Number of alleles** |
| --- | --- |
| Pgu 007 | 56 |
| Pgu 009 | 17 |
| Pgu 010 | 50 |
| Pgu 012 | 30 |
| Pgu 014 | 11 |
| Pgu 016 | 36 |
| Pgu 017 | 38 |
| Pgu 019 | 18 |
| Pgu 021 | 26 |
| Pgu 022 | 25 |
| Pgu 025 | 34 |
| Pgu 027 | 21 |
| Pgu 029 | 23 |
| Pgu 032 | 13 |
| Pgu 034 | 60 |
| Pgu 036 | 35 |
| Pgu 038 | 25 |
| Pgu 041 | 13 |
| Pgu 042 | 17 |
| Pgu 044 | 37 |
