# Supplementary material for: High Risks of Losing Genetic Diversity in an Endemic Mauritian Gecko: Implications for Conservation
Source: PLoS One. 2014 Jun 25;9(6):e93387. doi: 10.1371/journal.pone.0093387 (PMC4070904; doi:10.1371/journal.pone.0093387)
Supplement: Table S7 — The mean probability of no migration and migration among ten subpopulations of Phelsuma guimbeaui obtained in GENECLASS2. (DOC) [file pone.0093387.s007.doc]

**Table S7.** The mean probability of no migration and migration among ten subpopulations of *Phelsuma guimbeaui* obtained in GENECLASS2.

| **Sample site** | **No migration** | **Migration** |
| --- | --- | --- |
| L1 | 1 | 0 |
| L2 | 0.929 | 0.071 to L6 |
| L3 | 1 | 0 |
| L4 | 0.957 | 0 |
| L5 | 0.931 | 0.069 to L6 |
| L6 | 0.966 | 0.034 to L5 |
| L7 | 0.957 | 0.043 to L6 |
| L8 | 0.923 | 0.038 to L9; 0.038 to L10 |
| L9 | 0.962 | 0.038 to L8 |
| L10 | 1 | 0 |
| Average | 0.963 | 0.030 |
