# Supplementary material for: High Risks of Losing Genetic Diversity in an Endemic Mauritian Gecko: Implications for Conservation
Source: PLoS One. 2014 Jun 25;9(6):e93387. doi: 10.1371/journal.pone.0093387 (PMC4070904; doi:10.1371/journal.pone.0093387)
Supplement: Table S8 — Haplotype distribution and frequency in 80 individuals from 13 subpopulations of Phelsuma guimbeaui. (DOC) [file pone.0093387.s008.doc]

**Table S8. Haplotype distribution and frequency in 80 individuals from 13 subpopulations of *Phelsuma guimbeaui*.**

| **Haplotype** | **Subpopulations** | **Frequency** | **% frequency** |
| --- | --- | --- | --- |
| Hap_1 | L1, L3 | 6 | 7.5 |
| Hap_2 | L1, L3 | 4 | 5.0 |
| Hap_3 | L3, L4, L5, L6, L11 | 15 | 18.8 |
| Hap_4 | L2, L12 | 7 | 8.8 |
| Hap_5 | L2 | 1 | 1.3 |
| Hap_6 | L4 | 2 | 2.5 |
| Hap_7 | L4 | 1 | 1.3 |
| Hap_8 | L4, L13 | 4 | 5.0 |
| Hap_9 | L4, L10 | 6 | 7.5 |
| Hap_10 | L12 | 1 | 1.3 |
| Hap_11 | L12 | 1 | 1.3 |
| Hap_12 | L5 | 2 | 2.5 |
| Hap_13 | L5 | 2 | 2.5 |
| Hap_14 | L5 | 2 | 2.5 |
| Hap_15 | L6 | 1 | 1.3 |
| Hap_16 | L7, L10 | 6 | 7.5 |
| Hap_17 | L7 | 1 | 1.3 |
| Hap_18 | L7 | 1 | 1.3 |
| Hap_19 | L8 | 7 | 8.8 |
| Hap_20 | L8 | 1 | 1.3 |
| Hap_21 | L9 | 5 | 6.3 |
| Hap_22 | L9 | 1 | 1.3 |
| Hap_23 | L9 | 1 | 1.3 |
| Hap_24 | L10 | 1 | 1.3 |
| Hap_25 | L10 | 1 | 1.3 |
